# Seasonal and interannual variations of soil heterotrophic respiration and autotrophic respiration in subtropical forests of southeast China: independent process-based models

**DOI:** 10.1186/s40562-025-00399-1

**Published:** 2025-06-21

**Authors:** Yibo Yan, Xiujun Wang, Georg Wohlfahrt, Ni Huang

**Affiliations:** 1 Faculty of Geographical Science, Beijing Normal University, Beijing, 100875 China; 2 Universität Innsbruck, Institut für Ökologie, 6020 Innsbruck, Austria; 3 State Key Laboratory of Remote Sensing Science, Aerospace Information Research Institute, Chinese Academy of Sciences, Beijing, 100094 China

**Keywords:** Soil heterotrophic respiration, Soil autotrophic respiration, Seasonality, Inter-annual variations, Process-based models, Subtropical forests

## Abstract

Soil respiration consists of two distinctive components: heterotrophic respiration (decomposition of soil organic matter) and autotrophic respiration (CO_2_ production from root system), which are driven by different factors. However, the absence of process-based independent models for the two components hampers our ability to accurately quantify and understand the key carbon sources on land. Here, we used observational data to develop independent models for both soil heterotrophic and autotrophic respiration in the subtropical forests of southeast China, and validated the models using independent field data. Applying the validated models, we estimated both soil heterotrophic respiration and autotrophic respiration at three forest sites over 2002–2022. Our results showed higher annual rates of heterotrophic respiration (0.8–1.6 g C m^−2^ day^−1^) than autotrophic respiration (0.5–0.8 g C m^−2^ day^−1^). There was significant seasonality and inter-annual variability in both components, with larger variations in autotrophic respiration. The inter-annual variation was strongest in the winter season for both two respiration components. We found a significant (*p* < 0.01) increasing trend in autotrophic respiration (with a slope of 2.0–6.0 g C m^−2^ year^−2^) but not in heterotrophic respiration over 2002–2022. There was also a significant increasing trend in total soil respiration (3.4–6.2 g C m^−2^ year^−2^ for slopes), indicating an increasing role of autotrophic respiration. Our results reveal the dynamic nature of soil heterotrophic respiration and autotrophic respiration and their differing responses to environmental changes, emphasizing the need for independent models to improve the understanding of soil CO_2_ sources in a changing climate.

**Supplementary Information:**

The online version contains supplementary material available at 10.1186/s40562-025-00399-1.

## Introduction

Soil respiration is a major CO_2_ source in the terrestrial ecosystem, having a significant influence on atmospheric CO_2_ concentration (Bond-Lamberty and Thomson 2010; Watts et al. 2021). Globally, soil respiration accounts for more than half of the total respiration in forest ecosystems (Litton and Giardina 2008; Zhang et al. 2022), which primarily consists of heterotrophic respiration and autotrophic respiration. While there is a large similarity in their seasonal variation, there are many differences between soil heterotrophic and autotrophic respiration in terms of spatial and temporal variations and regulating mechanisms (Jian et al. 2022b; Yang et al. 2018). Therefore, it is critical to evaluate the responses of both heterotrophic and autotrophic respiration to the changes of environmental conditions for better understanding the forest carbon sources/sinks.

Soil heterotrophic respiration, a process of microbial decomposition of soil organic matter (SOM), is influenced by the quantity and quality of SOM (Hursh et al. 2017) and environmental conditions (such as temperature and moisture) that determine substrate accessibility and microbial activity (Jiang et al. 2013). Soil autotrophic respiration represents CO_2_ production from roots, mycorrhizae, and other micro-organisms in the rhizosphere (Bond-Lamberty et al. 2004), which is related to plant’s growth and biomass (Diao et al. 2022). There is evidence of large differences in spatial distribution between soil heterotrophic and autotrophic respiration, e.g., smaller contribution of soil autotrophic respiration to total soil respiration in tropical and temperate forests than in arid and cold regions (Jian et al. 2022b).

There is also evidence of considerable differences in temporal variation between soil heterotrophic and autotrophic respiration in terrestrial ecosystem (Bhanja et al. 2022; Hopple et al. 2023; Yu et al. 2015). For example, soil autotrophic respiration increased more rapidly than heterotrophic respiration during the growing season in some temperate and subtropical forests (Lee et al. 2010; Shi et al. 2012). A global scale meta-analysis revealed a significant increasing trend in heterotrophic respiration, but not in autotrophic respiration during 1987–2016 (Lei et al. 2021), whereas an increasing trend was shown in both heterotrophic and autotrophic respiration since 1980 s in modeling studies (Tang et al. 2020, 2021). There are also differences in the responses of heterotrophic and autotrophic respiration to changes in climate conditions in subtropical forests, e.g., a clear increase only in heterotrophic respiration under warming (Liu et al. 2019b), and a significant decrease only in autotrophic respiration under reduced rainfall (Huang et al. 2018). On the other hand, there is evidence of significant impacts of climate variability such as El Niño–Southern Oscillation (ENSO) on soil respiration in tropical and subtropical regions (Poulter et al. 2014; Zarzosa et al. 2020).

Due to the limited availability of field data, modeling approaches have been used to estimate soil respiration in various terrestrial ecosystems (Hashimoto et al. 2015; Huang et al. 2015). However, most statistical models only simulate total soil respiration, without separation into heterotrophic and autotrophic respiration, which could cause uncertainties in estimated rates of soil respiration (Huang et al. 2020; Jian et al. 2020; Yu et al. 2010). Indeed, there is a mismatch in the seasonality between modeled and measured total soil respiration in some forests, e.g., evergreen broadleaf forest and mixed forest (Fig. 5 in Jian et al. 2020), which could be due to the different responses of heterotrophic and autotrophic respiration to environmental drivers. Although soil heterotrophic and autotrophic respiration are considered separately in many process-based models, there is a lack of calibration/validation of soil respiration in most models (Chen et al. 2023; Lawrence et al. 2019; Mauritsen et al. 2019), and there are considerable differences in modeled rates of soil heterotrophic and/or autotrophic respiration among models (Lu et al. 2021). There is thus a need of advanced soil respiration models with improved parameterizations of both heterotrophic and autotrophic respirations for better understanding of the terrestrial carbon cycle.

Subtropical forests in southeast China, a major carbon sink, have shown a greening trend since the 1980 s, attributed to land use and/or climate changes (Zhu et al. 2016). Given the different responses of heterotrophic and autotrophic respiration to the changes of climate and vegetation conditions, one may hypothesize that there would be large differences in the temporal variations between soil heterotrophic respiration and autotrophic respiration in subtropical forests. To test this hypothesis, we conducted a process-based modeling study at representative forest sites in southeast China, where data of soil heterotrophic respiration and autotrophic respiration together with other relevant parameters were available. The objectives of this study are twofold: (1) to search and gather observational data to develop and refine independent models of soil heterotrophic respiration and autotrophic respiration for subtropical forests and (2) to apply the validated models to estimate soil heterotrophic respiration and autotrophic respiration at three study sites over 2002–2022, and analyze the differences in the seasonality and inter-annual variability between the two components. This study provides crucial insights into soil carbon fluxes, aiming to improving carbon cycle models and our understanding of ecosystem responses to climate change. Independent models for heterotrophic and autotrophic soil respiration have the potential to further improve soil carbon modules in terrestrial carbon cycle models.

## Materials and methods

### Field data

**Fig. 1:**
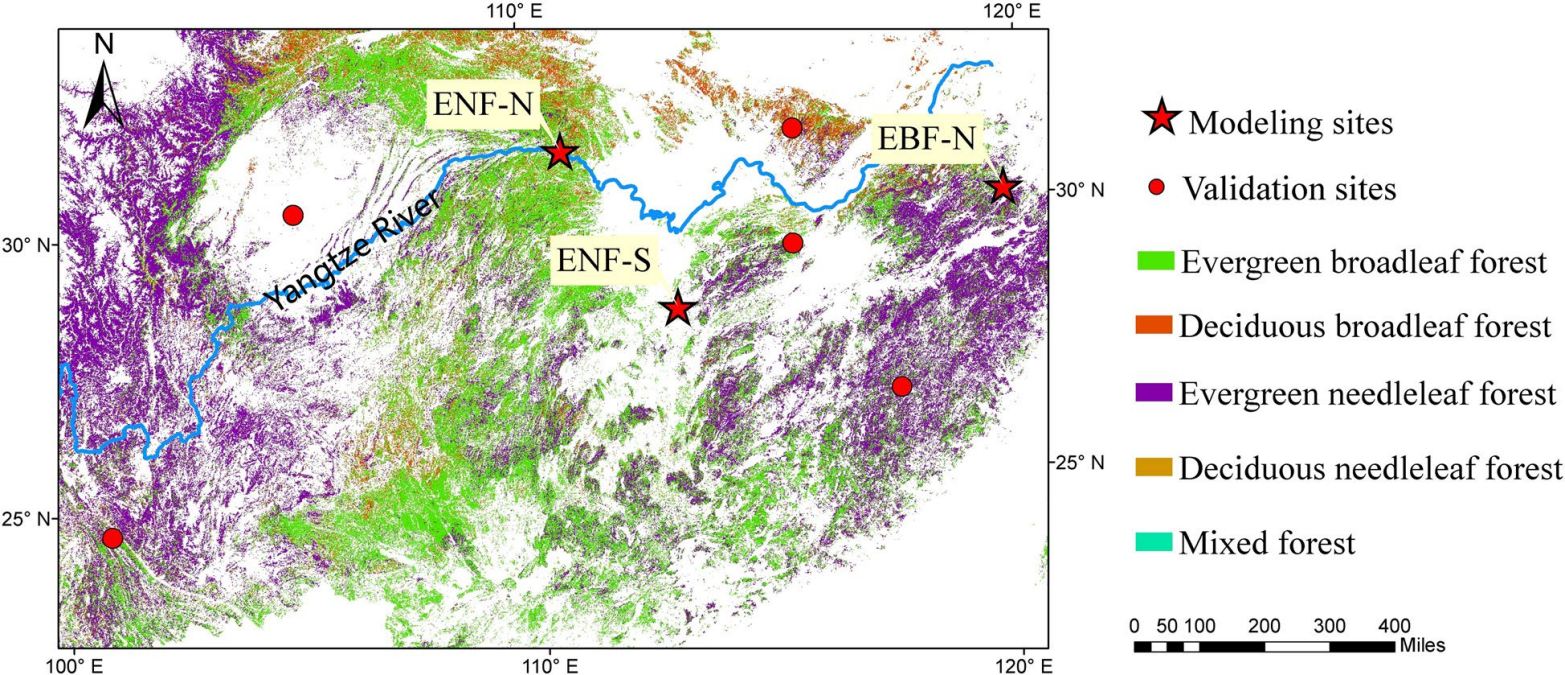
Map of subtropical forests and locations of selected sites in southeast China. The different colors represent the distributions of main forest types in the region (https://maps.elie.ucl.ac.be/CCI/viewer/)

We conducted a literature search for field data collection over entire China’s subtropical forest. Three sites were selected for model calibration and selection (modeling sites in Fig. 1), based on four criteria: (1) continuous field measurements (at least over the growing season) of soil heterotrophic respiration and autotrophic respiration together with soil temperature and soil moisture; (2) using the same method (e.g., trenching) to separate the two respiration components and with the removal of living vegetation and litter during the measurement; (3) reliable field measurements with long enough (>6 months) standing period after trenching; and (4) reports of soil organic carbon (SOC), total nitrogen (TN) and other soil properties. All three sites locate in southeast China, where evergreen needle-leaf forest (ENF, *Quercus variabilis*) and evergreen broadleaf forest (EBF, *Pinus massoniana*) are the main forest types (Fig. 1), which are named as EBF-N (in Zhejiang), ENF-N (in Hubei) and ENF-S (in Hunan). The ENF-N site has the highest elevation (1225 m), the lowest temperature in summer (20.8 °C) and annual precipitation (1125 mm year^−1^). The ENF-S and EBF-N sites are situated at much lower elevation (< 150 m), with higher temperature in summer (> 25 °C) and precipitation (>1400 mm year^−1^). The main soil type is Acric Umbrisol at EBF-N, Leptic Luvisol at EBF-N and Haplic Acrisol at ENF-S based on WRB (2022). The EBF-N site has highest SOC (30.9 g kg^−1^) and TN (1.9 g kg^−1^), followed by ENF-N (SOC 22.7 g kg^−1^ and TN 1.1 g kg^−1^), and lowest at ENF-S (SOC 8.4 g kg^−1^ and TN 0.8 g kg^−1^).

For model validation, we identified five independent sites (i.e., validation sites in Fig. 1) that met the criteria of continuous measurements of heterotrophic or autotrophic respiration using the trenching method and with soil properties reported. The validation sites (with data of 92 heterotrophic respiration and 34 autotrophic respiration values) were distributed across southeast China, representing evergreen broadleaf and needleleaf forests, and mixed forests.

We used the Origin 2021 software (OriginLab 2021) (by manual digitization) to obtain the observational data from the published articles (He et al. 2023; Hu et al. 2018; Lei et al. 2018), which included total soil respiration, heterotrophic respiration and/or autotrophic respiration, together with soil temperature, soil moisture and SOC. Rates of total soil respiration (with roots) and heterotrophic respiration (without roots) were collected in the morning (local time, 9:00–11:00 am) every 2–4 weeks (mostly or bi-weekly) using an automated soil respiration system (LI-8100, LI-COR, USA). Heterotrophic respiration was measured using the trenching methods. Autotrophic respiration was calculated by subtracting heterotrophic respiration from total soil respiration. Soil temperature and moisture were measured at 5 cm using the LI-8100’s soil temperature and humidity probes. We conducted data quality control and removed abnormal data, e.g., heterotrophic respiration being greater than total soil respiration, and observations collected immediately after rainfall.

### Satellite data

For the purpose of model calibration and application, we downloaded and processed satellite-derived data of land surface temperature (LST), soil moisture (SM), leaf area index (LAI) and gross primary productivity (GPP) over 2002–2022. We selected the nighttime LST (mean value of 10:30 pm and 1:30 am) from MOD11A2 (daily, 1 km by 1 km) (Wan et al. 2021), due to its good correlation with in-situ soil temperature measurements (Fig. S1), which was consistent with the findings of Huang et al. (2020) and Yan et al. (2020). The SM data (daily, 1 km by 1 km) used was produced by Zheng et al. (2023b), which showed good agreement with field data at seasonal to inter-annual timescales (Zheng et al. 2023a). The LAI data (8 days, 500 m by 500 m) was from MOD15A2H (Myneni et al. 2021), which was calculated by an algorithm with a main Look-up-Table (LUT) and the back-up algorithm using empirical relationships between the normalized difference vegetation index (NDVI) and canopy LAI. The GPP data (8 days, 500 m by 500 m) was from MOD17A2H (Running et al. 2021), which was calculated by the Carnegie–Ames–Stanford Approach (CASA) (Running et al. 2004; Zhao et al. 2005). Previous studies demonstrated that the MODIS LAI and GPP products had good accuracy for subtropical forests (Huang et al. 2019a; Li et al. 2017; Zhao et al. 2020b).

Due to the difference between field data and satellite data, we converted satellite LST and SM to soil temperature and moisture at 5 cm based on their statistical relationships (Table S1). For the temporal match, we used linear interpolation to derive the satellite data at observational time of the selected sites. For the spatial match, we took necessary steps to extract satellite data at site scale. First, we determined the locations of the site pixel and its surrounding 8 pixels on Google Earth (https://www.google.com/maps), and identified ‘qualified’ pixels (with forest coverage > 70%) (Huang et al. 2019b; Yan et al. 2016). Second, we filled missing values and ‘corrected’ those pixels with low accuracy (due to the influence of cloud and rain), using the method of generative adversarial network and improved Savitzky–Golay filter (GAN and S–G, an improved technology for interpolating low-quality satellite LAI) (Huang et al. 2021), and then calculated the mean values from the ‘qualified’ pixels as the site value (Fig. S2). To evaluate the derived LAI/GPP data from MODIS, we compared them with GLASS data and found a good match in terms of seasonality and/or magnitude (see Fig. S3). We did not use GLASS data for model “forcing” because of its limitation over time range (i.e., 2000–2020) (Ma and Liang 2022).

### Development of soil heterotrophic and autotropic respiration models

#### Heterotrophic respiration model

Following Gu et al. (2004), soil heterotrophic respiration (HR) can be generally expressed as a function of *SOC* and soil temperature (*T*) that regulates microbe activity:

1 $$\text{HR}=\alpha \cdot SOC\cdot \text{exp}(\beta T)$$

where $\alpha$ is decomposition rate of unit SOC, and $\beta$ a constant which governs the temperature response.

Considering the impacts of soil moisture (M) on various biochemical processes (e.g., microbial activity and SOM stability) and thus complex effects on HR (Liu et al. 2019c; Wang et al. 2019), we test three different model structures:

2 $${\text{HR}}_{1}=SOC\cdot \left(\alpha +\gamma M\right)\cdot \text{exp}(\beta T)$$

3 $${\text{HR}}_{2}=\alpha \cdot SOC\cdot \left(\frac{M}{M+{k}_{M}}\right)\cdot \text{exp}(\beta T)$$

4 $${\text{HR}}_{3}=\alpha \cdot SOC\cdot \text{min}\left(\frac{T}{T+{k}_{T}} , \frac{M}{M+{k}_{M}}\right)\cdot \text{exp}(\beta T)$$

where $\gamma$ is a constant which governs the moisture response, ${k}_{T}$ and ${k}_{M}$ Michaelis–Menten constants (set as the half of the maximum) for soil temperature and moisture, respectively. HR_1_ is based on studies of Yang et al. (2021) and Xiong et al. (2021), and HR_2_ on an earlier report of Jiang et al. (2013). HR_3_ is based on the theory of limiting influence of soil temperature and moisture on heterotrophic respiration (Liu et al. 2019c; Sur et al. 2020). The parameters ($\alpha$, $\gamma$ and$\beta$) are calculated by the least squares method using the software Origin 2021 (OriginLab 2021).

##### Autotrophic respiration model

According to Amthor (2000) and Fang et al. (2020), soil autotropic respiration (AR) conceptually consists of maintenance (Rm) and growth respiration (Rg):

5 $$\text{AR}=\text{Rm}+\text{Rg}$$

Maintenance respiration Rm is assumed to be a function of fine root biomass (FRB, calculated as *P*_*r_l*_* LAI*/*SLA*) and soil temperature *T*:

6 $$\text{Rm}={P}_{r\_l}\cdot \frac{LAI}{SLA}\cdot \delta \cdot {\varepsilon }^{\frac{T-20}{10}}$$

where ${P}_{r\_l}$ is the fine root-to-leaf ratio of biomass, fixed at 1.1 and 1.2 for EBF and ENF, respectively, and $SLA$ the projected leaf area per kg C, fixed at 25.9 and 14.1 for EBF and ENF, respectively (White 2000). $\delta$ is a parameter representing the maintenance respiration at 20 °C, and $\upvarepsilon$ a constant which governs the temperature response.

Following Cannell and Thornley (2000) and Ryan (1991), plant growth respiration is assumed to constitute 25% of net primary productivity (NPP). Accordingly, root growth respiration Rg is calculated as follows:

7 $$\text{Rg}=0.25\cdot {P}_{r\_t}\cdot NPP$$

where ${P}_{r\_t}$ is the portion of NPP allocated to the root system, set to 0.15 according to meta-analysis by Jian et al. (2022a). *NPP* is calculated by subtracting total autotrophic respiration (TAR) from *GPP*, and TAR is calculated as

8 $$\text{TAR}=\text{AR}/{P}_{r\_t}$$

By combining Eqs. (5)–(8), we obtain the following equation for AR:

9 $$\text{AR}=0.8{\cdot P}_{r\_l}\cdot \frac{LAI}{SLA}\cdot \delta \cdot {\varepsilon }^{\frac{T-20}{10}}+0.03\cdot GPP$$

To determine the parameter values for $\delta \text{ and }\varepsilon ,$ we compare two methods: (1) using the values in the Biome-Property-Look-Up-Table (BPLUT) (White 2000) and (2) using the Levenberg–Marquardt (LM) optimization algorithm by the software Origin 2021 (OriginLab 2021).

### Model selection, evaluation and validation

Model selection, evaluation and validation were based on assessments of four statistical indicators: coefficient (R^2^) of regression through the origin with its significance, root mean squared error (RMSE), normalized standard deviation (NSD) and Akaike information criterion (AIC). In addition, we also considered the models’ capacity of simulating the seasonality (represented by the linear regression slope between modeled and observed rates) of soil heterotrophic and autotropic respiration.

The AIC is calculated according to Shi and Tsai (1998):

10 $$\text{AIC}=2\text{k}+\text{n}\cdot \text{ln}(\text{RSS}/\text{n})$$

where n is the number of samples, and k is the number of model parameters. RSS (residual sum of squares) is the sum of squares of residuals between modeled and observed values.

### Analysis of driving factors of inter-annual variation in heterotrophic and autotrophic respiration

To calculate the relative and combined contributions of soil temperature and moisture (soil temperature, FRB and GPP) to the changes in soil heterotrophic respiration (autotrophic respiration), we used a linear perturbation analysis similar to Wilson and Baldocchi (2000) with a multivariate Taylor’s expansion (ignoring the higher-order terms):

11 $$\delta \text{HR}=\frac{\delta HR}{\delta T}dT+\frac{\delta HR}{\delta M}dM$$

12 $$\delta \text{AR}=\frac{\delta AR}{\delta T}dT+\frac{\delta AR}{\delta FRB}dFRB+\frac{\delta AR}{\delta GPP}dGPP$$

where $\delta \text{HR}$ and $\delta \text{AR}$ are the changes of annual mean heterotrophic or autotrophic respiration relative to the 2002 rates.

In addition, we also employed correlation analysis to examine the linear relationships between soil heterotrophic respiration (autotrophic respiration) and soil temperature and soil moisture (soil temperature, FRB, and GPP).

## Results

### Model selection, evaluation and validation

As shown in Fig. S4, all three heterotrophic respiration models showed capacity in reproducing the seasonality of heterotrophic respiration (i.e., close-to-1 slopes) and significant correlation between modeled and observed rates (R^2^ = 0.74–0.92, *p* < 0.01). We selected HR_3_ model because of its lower average RMSE (0.186 g C m^−2^ day^−1^) and AIC (−47.9) at three sites compared to the other two models (Table S2). Furthermore, the HR_3_ model demonstrated improved accuracy in simulating summer respiration rates (Fig. S4a, c), primarily because it incorporated temperature–moisture interactions that constrained microbial activity. This mechanistic improvement allowed HR_3_ to better represent the non-monotonic response of HR to summer temperature and moisture conditions—a key ecological process that the other two models failed to capture. For autotropic respiration models (Fig. S5), both parametric methods provided good outcomes, i.e., significant correlation (*p* < 0.01), low RMSE (0.19–0.37 g C m^−2^ day^−1^) and close-to-1 NSD (Table S3). Although AR_LM_ showed a slightly lower AIC and RMSE at EBF-N, we selected AR_BPLUT_ because of its better performance in the seasonality (indicated by a closer-to-1 slope and NSD) at all three sites. Moreover, the BPLUT method did not require site-specific calibration. The selected models and parameters are shown in Table S4.

**Fig. 2:**
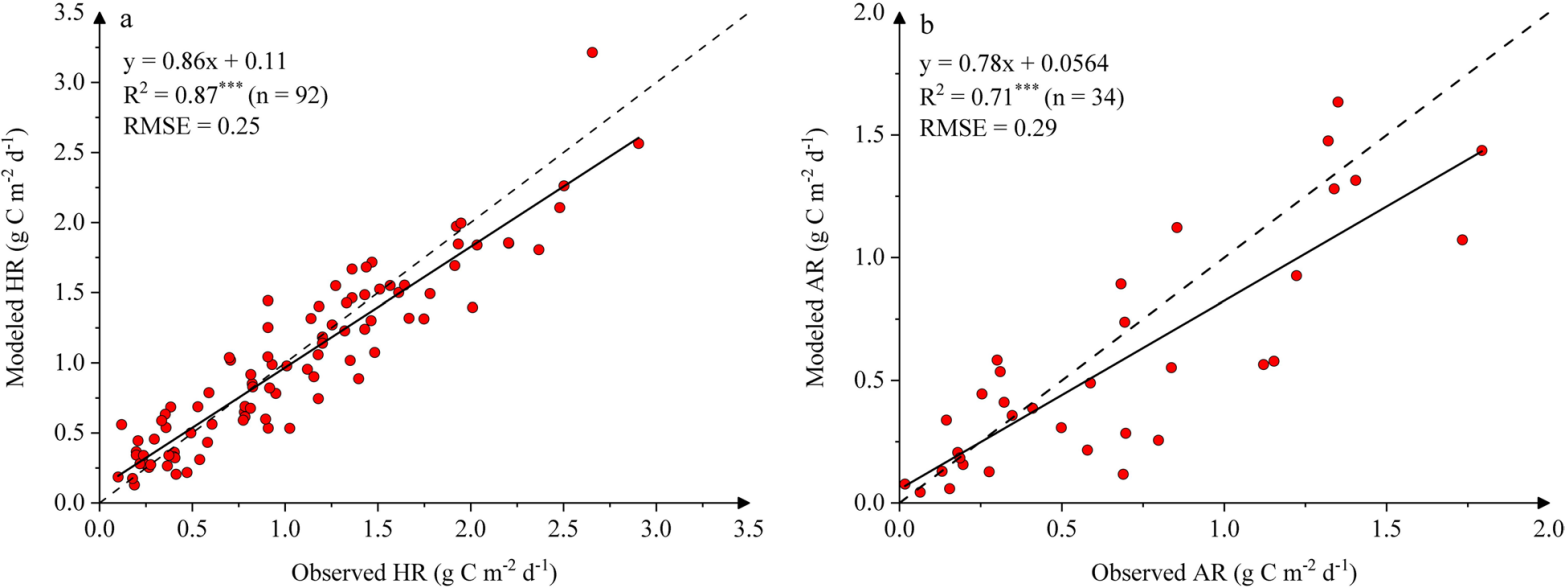
Correlation between modeled and observed **a** heterotrophic (HR) and **b** autotrophic respiration (AR) at validation sites. The solid line represents the linear regression fit between simulations and observations, while the dashed 1:1 line denotes perfect agreement. Three asterisks indicate the significance at *p* < 0.001

We used other field data from five independent sites to validate the models of HR_**3**_ and AR_BPLUT_. Clearly, there was a significant correlation (*p* < 0.001) between modeled and observed rates for both heterotrophic respiration and autotrophic respiration (Fig. 2). It appeared that modeled rates of heterotrophic respiration had better agreement with observational data compared to autotrophic respiration, as indicated by a higher correlation coefficient, a closer-to-1 slope, and lower RMSE. The bias between modeled and observed rates will be discussed in"Model parameters, uncertainties and limitations"section.

### Seasonality of driving factors and simulated heterotrophic and autotrophic respiration

**Fig. 3:**
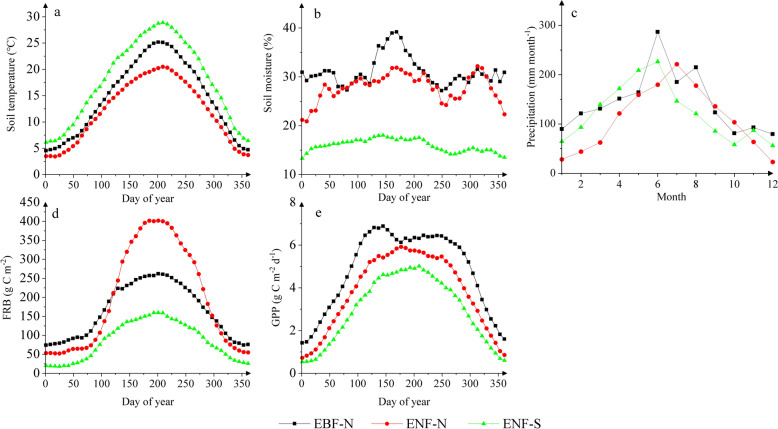
Seasonal variation of **a** soil temperature, **b** soil moisture, **c** precipitation, **d** fine root biomass (FRB), and **e** GPP at three sites

Figure 3 shows clear seasonality in soil temperature, soil moisture, precipitation, calculated FRB, and satellite-derived GPP at the three modeling sites. The seasonal amplitude (summer–winter) of soil temperature was larger at ENF-S (20 °C) and EBF-N (18.1 °C) than at ENF-N (15.3 °C) (Table S5). Precipitation showed much greater seasonal amplitude at the two northern sites (132–161 mm month^−1^) than at the southern site (93 mm month^−1^). There was a considerable difference in the seasonal pattern between soil moisture and precipitation particularly at ENF-N, with two peaks (in June and November) of soil moisture, but only one peak (in June) of precipitation (Fig. 3b, c). Clearly, the seasonality was weaker in soil moisture than in precipitation. There was a large difference in seasonality of the calculated FRB among the three sites (Fig. 3d), showing a much greater seasonal amplitude at ENF-N (324 g C m^−2^) than at EBF-N and ENF-S (125–172 g C m^−2^) (Table S5). There was also a modest difference in satellite-derived GPP seasonality among the three sites (Fig. 3e), i.e., a slightly larger seasonal amplitude at ENF-N and EBF-N (4.3–4.5 g C m^−2^ day^−1^) than at ENF-S (4 g C m^−2^ day^−1^) (Table S5).

**Fig. 4:**
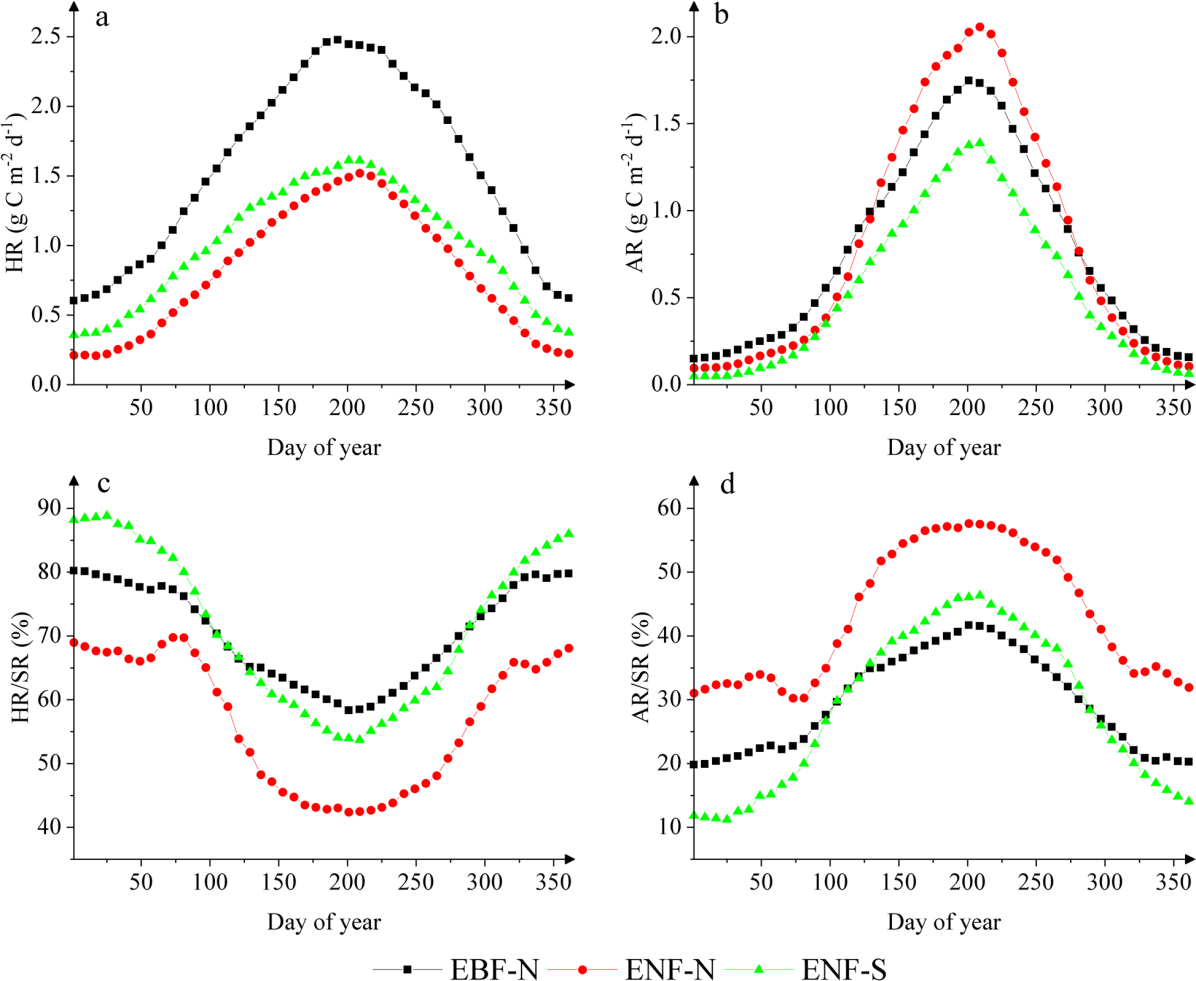
Seasonal variation of **a** heterotrophic respiration (HR), **b** autotrophic respiration (AR), the contribution of **c** HR to total soil respiration (HR/SR) and **d** AR to total soil respiration (AR/SR) at the three sites

A clear seasonality was seen in heterotrophic respiration with one peak in July at ENF-N and ENF-S and in June at EBF-N (Fig. 4a). Rates of heterotrophic respiration were higher at EBF-N than at the two ENF sites, with relatively smaller differences in January (0.6 vs. 0.2–0.4 g C m^−2^ day^−1^), than in July (2.5 vs. 1.5–1.6 g C m^−2^ day^−1^), indicating a much greater seasonal amplitude at EBF-N compared to the ENF sites (1.7 vs. 1.1–1.2 g C m^−2^ day^−1^) (Table S5). There was also a strong seasonality in autotrophic respiration with one peak in July at all three sites (Fig. 4b), showing higher annual mean rates at the two northern sites (0.8 g C m^−2^ day^−1^) than at ENF-S (0.5 g C m^−2^ day^−1^). The seasonal amplitude of autotrophic respiration was greatest at ENF-N (1.7 g C m^−2^ day^−1^), followed by EBF-N (1.4 g C m^−2^ day^−1^) and ENF-S (1.1 g C m^−2^ day^−1^) (Table S5). The “speed/velocity” of increase in spring and decrease in autumn was greater in autotrophic respiration than in heterotrophic respiration at all three sites (Fig. 4a, b), reflecting a much stronger seasonality in autotrophic respiration (C.V. = 70–85%) than in heterotrophic respiration (C.V. = 42–56%) (Table S5). As a result, the contribution of autotrophic respiration to total soil respiration (AR/SR) was significantly higher in summer (39–57%) than in winter (13–33%), whereas the contribution of heterotrophic respiration (HR/SR) was significantly higher in winter (67–87%) than in summer (43–61%) (Fig. 4c, d).

### Inter-annual variation of simulated heterotrophic and autotrophic respiration

**Fig. 5:**
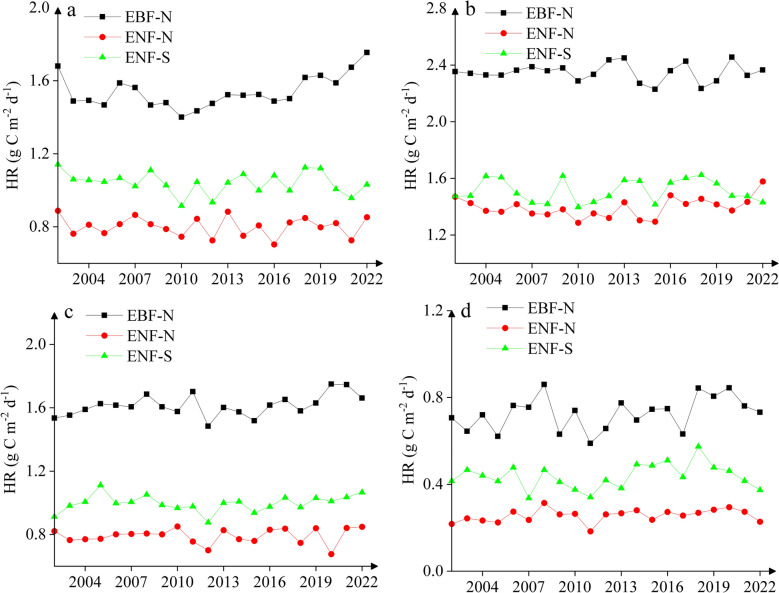
Inter-annual variation of heterotrophic respiration (HR) in **a** spring, **b** summer, **c** autumn and **d** winter over 2002–2022

Figure 5a illustrates a modest inter-annual variation of heterotrophic respiration in spring, showing no clear pattern at ENF-N and ENF-S, but an increasing ‘trend’ over the period of 2010–2022 at EBF-N. There was also a modest inter-annual variability of heterotrophic respiration in summer and autumn (Fig. 5b, c), showing considerable fluctuations in summer at ENF-S and in both seasons (after ~2010) at EBF-N and ENF-N. Heterotrophic respiration in winter revealed a strong inter-annual variation at all three sites (Fig. 5d), with the lowest rates in 2011 and an obvious decreasing ‘trend’ over 2020–2022 when double/triple La Niña events occurred. Overall, there was a greater detrended standard deviation (DSD) at EBF-N and ENF-S (0.06–0.08 g C m^−2^ day^−1^) than at ENF-N (0.03 g C m^−2^ day^−1^, Table S6).

**Fig. 6:**
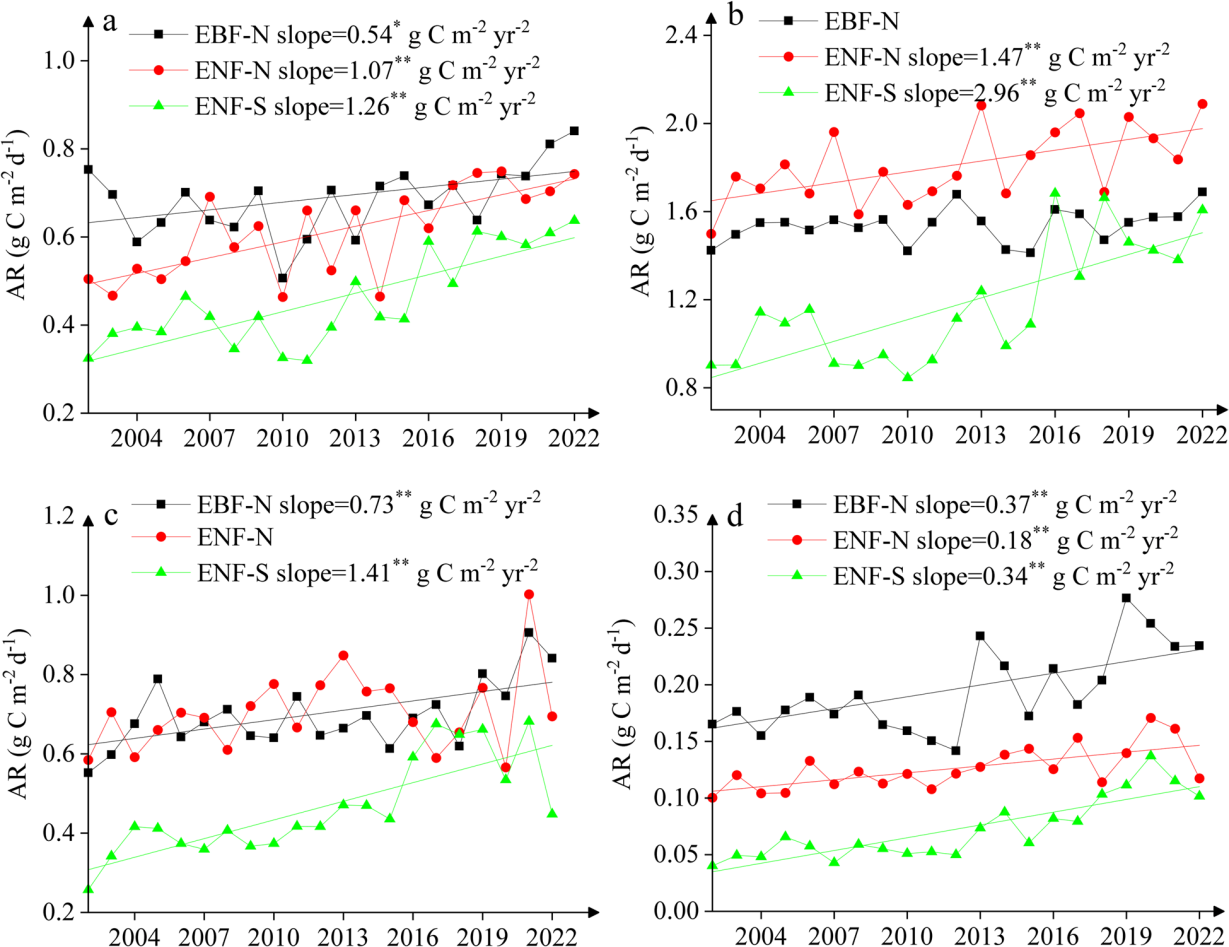
Inter-annual variation of autotrophic respiration (AR) in **a** spring, **b** summer, **c** autumn and **d** winter over 2002–2022. Straight lines denote significant trends and slopes indicating annual change rates. One asterisk and two asterisks indicate the significance at *p* < 0.05 and at *p* < 0.01, respectively

Autotrophic respiration showed strong fluctuations in spring particularly prior to 2016 at all three sites with the lowest rates in 2010/2011, but smaller differences over 2016–2022 among the three sites (Fig. 6a). There was a significant increasing trend of autotrophic respiration in spring over 2002–2022 at all three sites, with a much greater slope at the two ENF sites (1.07–1.26 g C m^−2^ year^−2^, *p* < 0.01) than at the EBF site (0.54 g C m^−2^ year^−2^, *p* < 0.01). Autotrophic respiration also showed strong fluctuations in summer with relative high rates over 2012–2022 particularly at the two ENF sites (Fig. 6b), which had a greater DSD (0.14–0.18 g C m^−2^ day^−1^) than EBF site (0.07 g C m^−2^ day^−1^) (Table S6). There was also a significant increasing trend (*p* < 0.01) of autotrophic respiration in summer at ENF-S (2.96 g C m^−2^ year^−2^) and ENF-N (1.47 g C m^−2^ year^−2^). For the autumn and winter seasons, autotrophic respiration revealed a small fluctuation in the first decade but strong inter-annual variation in the recent decade at the three sites (Fig. 6c, d). Similar to heterotrophic respiration, there was also an obvious decreasing ‘trend’ in winter rates of autotrophic respiration over 2020–2022 when triple La Niña events occurred (Fig. 6d). There was a significantly increasing trend (*p* < 0.01) of autotrophic respiration at the EBF-N and ENF-S (0.73–1.41 g C m^−2^ year^−2^) in autumn, and at all three sites in winter (0.18–0.37 g C m^−2^ year^−2^) over 21 years. Overall, the inter-annual variation of autotrophic respiration was strongest in winter and weakest in summer, as indicated by normalized C.V. (NCV) values, i.e., 13–41% in winter and 4–21% in summer (Table S6).

**Fig. 7:**
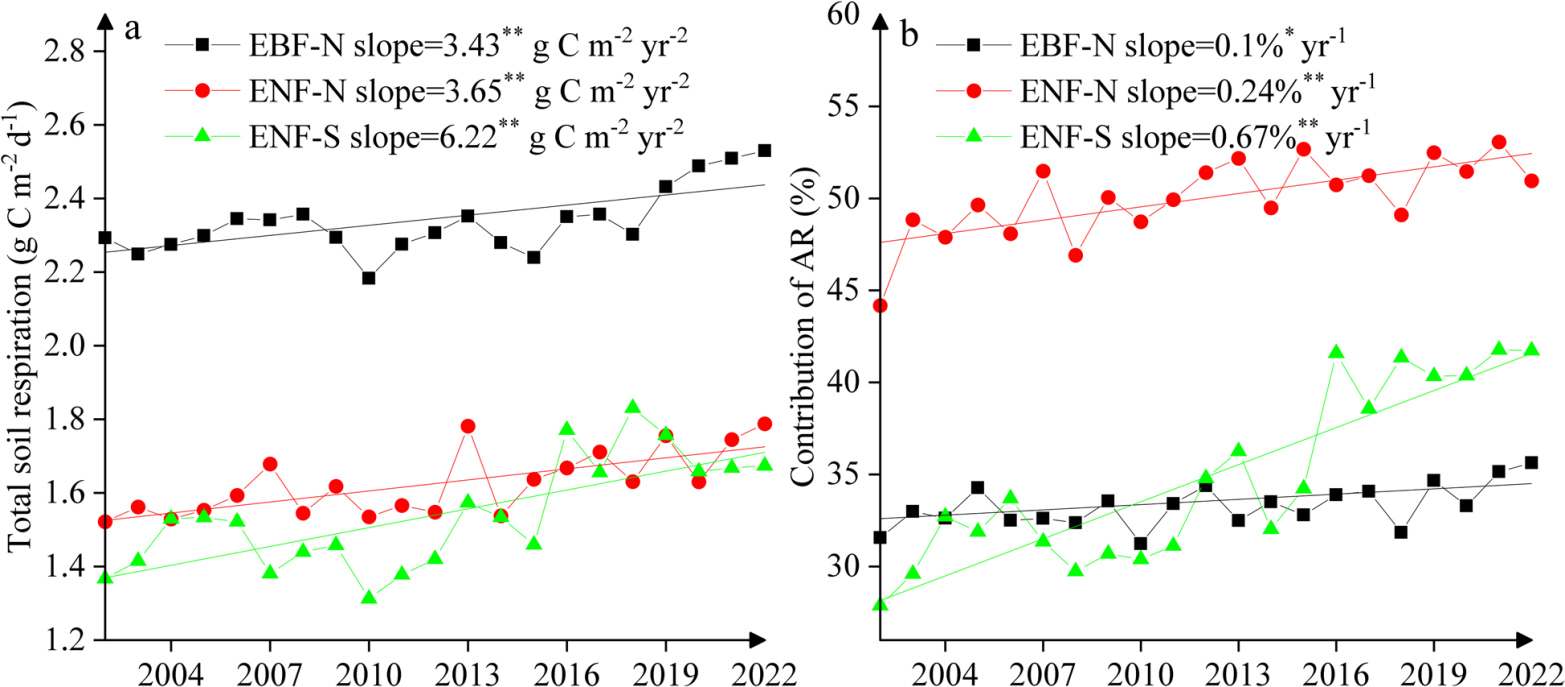
Inter-annual variation of **a** annual average of total soil respiration and **b** contribution of autotrophic respiration (AR) to total soil respiration over 2002–2022. Straight lines denote significant trends and slopes indicating annual change rates. One asterisk and two asterisks indicate the significance at *p* < 0.05 and at *p* < 0.01, respectively

Total soil respiration exhibited a considerable inter-annual variation at all three sites, with a significant increasing trend (*p* < 0.01) over 2002–2022 (Fig. 7a). The change rate (i.e., slope) was much greater at ENF-S (6.22 g C m^−2^ year^−2^) than at the two northern sites (3.43–3.65 g C m^−2^ year^−2^), mainly due to enhanced autotrophic respiration over 2016–2022 at ENF-S (Fig. 6). The contribution of autotrophic respiration to total soil respiration was much greater at ENF-N (50% ± 2%) than at EBF-N (33% ± 1%) and ENF-S (35% ± 5%) (Fig. 7b). There was also a significantly increasing trend (*p* < 0.01) in autotrophic respiration’ contribution at all three sites, with much greater slope at ENF-S (0.67% year^−1^) than other two sites (0.1–0.24% year^−1^).

### Driving mechanism of inter-annual variability of soil heterotrophic and autotrophic respiration

**Fig. 8:**
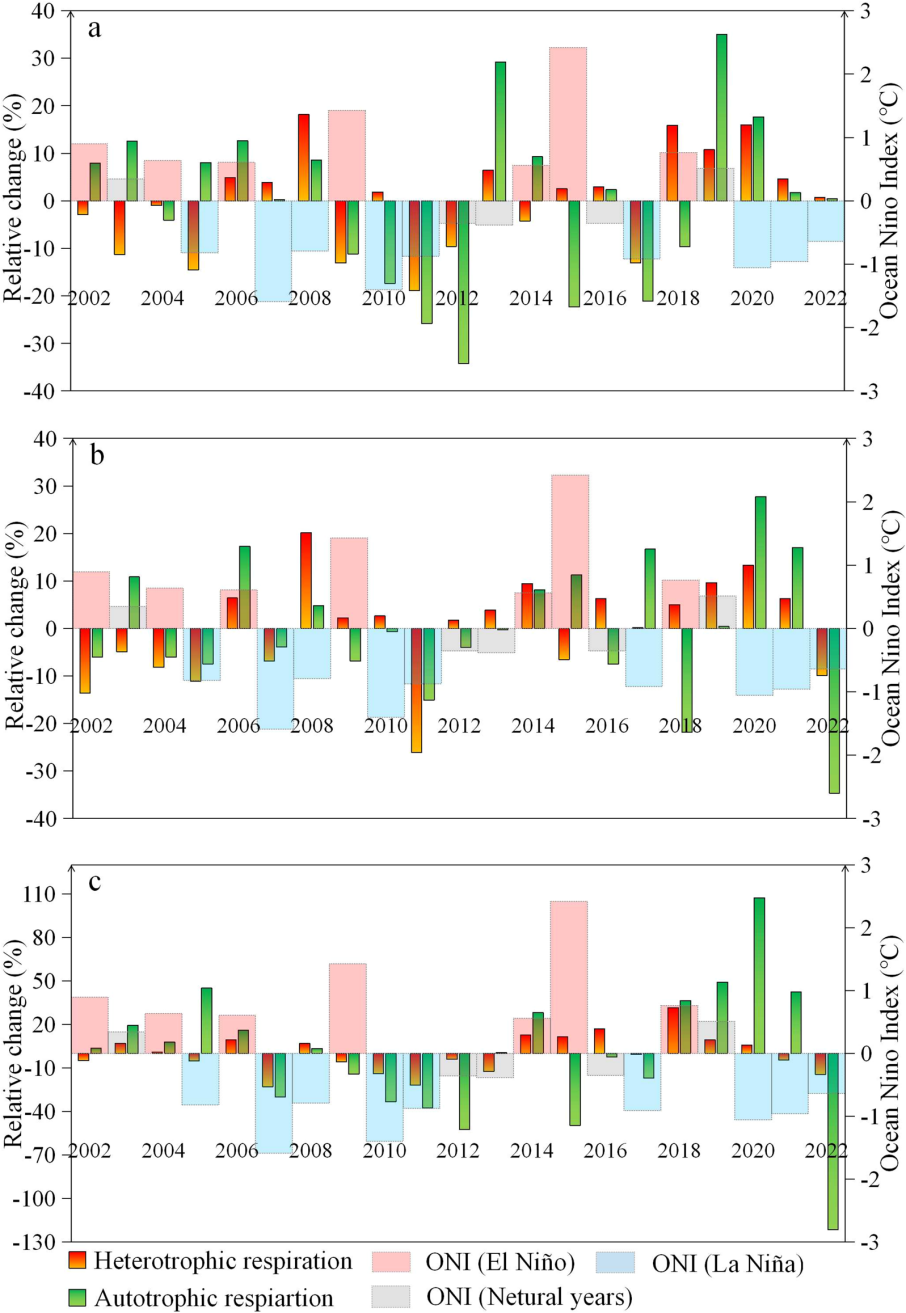
Relative changes of annual heterotrophic and autotrophic respiration and Ocean Nino Index (ONI) in winter at **a** EBF-N, **b** ENF-N and **c** ENF-S compared with the detrended-mean over 2002–2022

Given that both heterotrophic respiration and autotrophic respiration showed the largest inter-annual variation in winter (with lower rates during/following some La Niña events), we evaluated the relative changes (compared with the detrended-mean of all years) of the winter rates during ENSO events. Figure 8a, b shows a significant increase of wintertime heterotrophic respiration during both warm and cold ENSO events at the two northern sites, e.g., >15% increase during 2018–19 El Niño, and 2008–09 and 2020–21 La Niña events at EBF-N. For ENF-S, heterotrophic respiration showed an increase (5–30%) in five out of seven El Niño events and a decrease (5–25%) in seven out of nine La Niña events (Fig. 8c). As shown in Table S7, the detrended means of soil heterotrophic respiration were similar between El Niño and La Niña years at the two northern sites, but higher during El Niño (0.47 g C m^−2^ day^−1^) than during La Niña (0.4 g C m^−2^ day^−1^) events at the southern site (ENF-S). On average, heterotrophic respiration showed an increase of 4.4% during El Niño years and a decrease of 10.9% during La Niña years at ENF-S, compared with neutral years. Correlation analysis showed a significant positive correlation (*p* < 0.05) between the winter rate of heterotrophic respiration and the ENSO index–Oceanic Niño Index (ONI) only at ENF-S. The inter-annual variation of autotrophic respiration in winter appeared not to be influenced by ENSO events at all three sites (Fig. 8). For example, autotrophic respiration showed inconsistent responses to super La Niña events at the three sites, e.g., little change in 2007–08, 19% decrease in 2010–11, but 18% increase in 2020–21 at EBF-N. On average, there was a small difference in autotrophic respiration between El Niño and La Niña events, and the relative change to the mean of all years was <5% during both types of events at all three sites (Table S7).

**Fig. 9:**
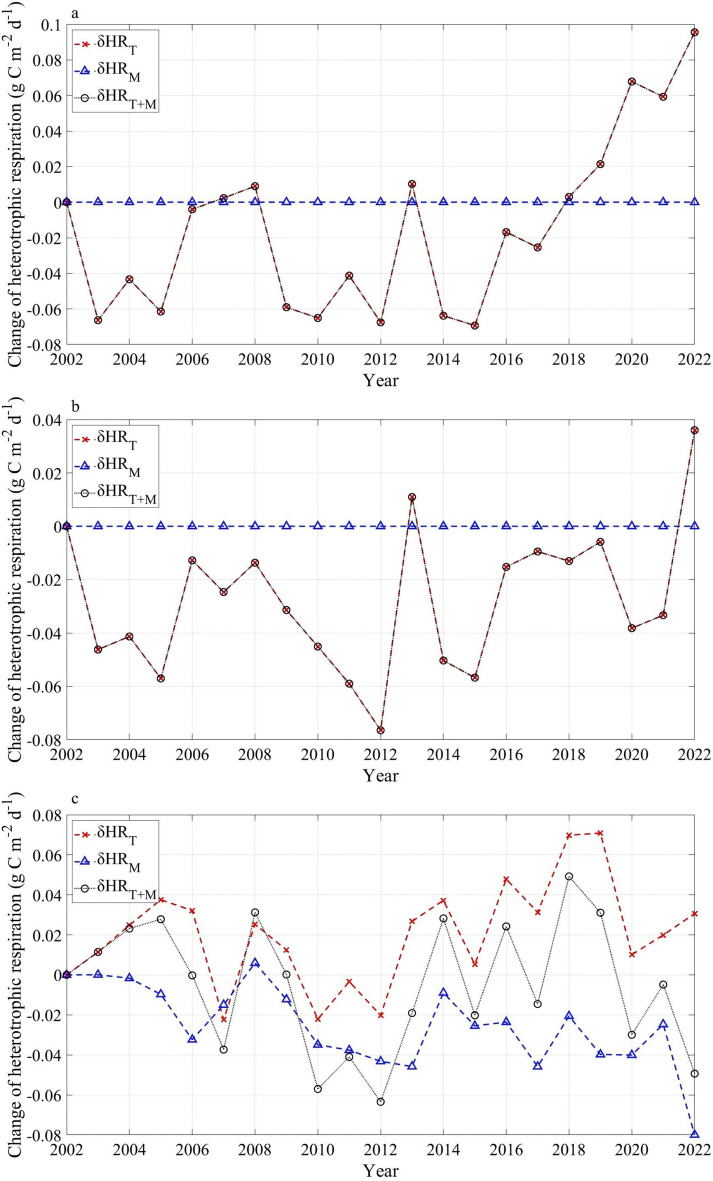
Heterotrophic respiration changes driven by soil temperature (δHR_T_) and oil moisture (δHR_M_) at **a** EBF-N, **b** ENF-N and **c** ENF-S over 2002–2022

**Fig. 10:**
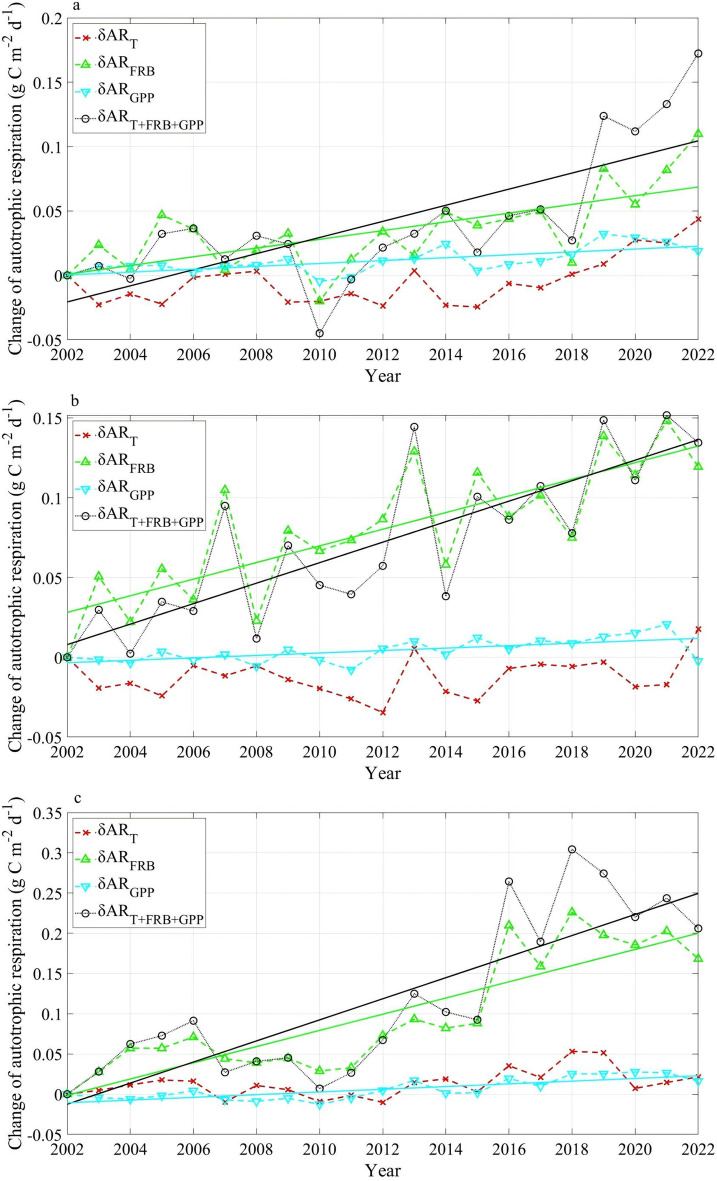
The autotrophic respiration changes driven by soil temperature (δAR_T_), fine root biomass (δAR_FRB_) and GPP (δAR_GPP_) at **a** EBF-N, **b** ENF-N and **c** ENF-S over 2002–2022. Straight lines denote a significantly increasing trend with the significance of *p* < 0.01

The linear perturbation analysis (Fig. 9) showed that the annual change of heterotrophic respiration was regulated exclusively by soil temperature over 2002–2022 at the two northern sites (EBF-N and ENF-N). For the southern site ENF-S, with relatively low soil moisture, soil moisture considerably dampened the temperature-driven change of heterotrophic respiration during years with high temperatures and reinforced the decline in heterotrophic respiration during cooler years (with 49% contribution to the change of heterotrophic respiration) (Fig. 9c, Table S8). Similarly, correlation analysis also showed that heterotrophic respiration had a significant positive relationship with soil temperature (*p* < 0.001) at all three sites especially in winter, and with soil moisture (*p* < 0.05) only in spring and summer at ENF-S (Table S7). On the other hand, there was a significant positive correlation (*p* < 0.05) between autotrophic respiration and soil temperature in all seasons only at EBF-N site. Soil temperature made small contributions (13–24%) to the annual changes of autotrophic respiration particularly at ENF-S (with the least contribution of 13%) (Table S8). Clearly, FRB made the largest contribution (54–78%) to the annual changes of autotrophic respiration at all three sites (Fig. 10a, Table S8). There was a significant increasing trend in FRB causing corresponding changes of autotrophic respiration, whose slope was similar to that from the combined contribution of all three factors (Fig. 10). Autotrophic respiration showed a significant positive correlation in all seasons with FRB (*p* < 0.01 or *p* < 0.001) at all three sites, but with GPP (*p* < 0.05) only at the two ENF sites (Table S9). GPP, showing a contribution of 6–22% to autotrophic respiration (Table S8), also exhibited a significant increasing trend (Fig. 10). Clearly, there was a larger contribution from GPP to autotrophic respiration’ change at EBF site (22%) than at ENF sites (6–9%) (Table S8).

## Discussion

### Model parameters, uncertainties and limitations

Our HR model was calibrated using site-specific data, revealing notable variations in key parameters across sites. The *α* values (0.07, 0.03, and 0.11 for the three sites), indicating the unit SOC decomposition rate, exhibited an inverse relationship with soil C/N ratios, consistent with the experimental finding that higher C/N ratios suppressed SOC decomposition (Wang et al. 2018; Yu et al. 2019). The *β* values (0.0236, 0.0594, and 0.039 for the three sites), determining temperature sensitivity of microbe activity, increased with decreasing mean annual soil temperature, aligning with observed strong temperature-sensitivities in colder environments (Chang et al. 2016; Kong et al. 2022; Xu et al. 2015). For the AR model, the parameters were forest-type dependent according to BPLUT (White 2000). The fine root-to-leaf biomass ratios (1.1 for broadleaf, 1.2 for needle leaf forests) closely matched field measurements in subtropical evergreen forests (1.05–1.28) (Wang et al. 2015; Zhang et al. 2015). The SLA values were fixed at 25.9 m^2^ kg⁻^1^ C (broadleaf) and 14.1 m^2^ kg⁻^1^ C (needle leaf) in our models, comparable to previous observations for needleleaf forest (13.5 m^2^ kg⁻^1^ C) (Qin and Shangguan 2019) and broadleaf forests (25.18 m^2^ kg⁻^1^ C) (Liu et al. 2019a) in subtropical China.

The independent validation demonstrated good overall consistency between model simulations and field data, though it revealed some minor discrepancies (Fig. 2). These biases likely stem from several sources. First, validation data came from continuous trenching observations. During these measurements, litter decomposition and dead root turnover in trenched plots (Savage et al. 2018; Zeng et al. 2016), along with suppressed rhizosphere microbial respiration from root severing (Han et al. 2011; Yan et al. 2015), might lead to seasonal over- or underestimation of soil heterotrophic respiration (Snell et al. 2014). Second, potential spatial-scale mismatches could arise, because our AR model relies on satellite-derived LAI and GPP to represent regional biomass and productivity. The inherent smoothing effect of gridded satellite data (Huang et al. 2014, 2017) might lead to underestimating both seasonal and spatial variations in autotrophic respiration. Figure 2b exemplifies this, showing the model systematically underestimates AR when observed values exceed 0.5 g C m⁻^2^ d⁻^1^. In addition, field measurements often lacked documentation of sampling positions relative to tree trunks (Zhao et al. 2021). This omission might be significant, as studies confirmed that distance-to-trunk gradients considerably modulate SOC stocks and root biomass distributions (Ohashi et al. 2015; Yang et al. 2004). These unaccounted spatial heterogeneities likely contributed to the modeled-observed discrepancies (Wang et al. 2017).

At the model level, our HR model incorporated key controlling factors including temperature, moisture, and SOC, but did not directly account for microbial biomass and community composition that was demonstrated to significantly influence SOC decomposition (Sáez-Sandino et al. 2023; Wang et al. 2018). While our AR model improved upon previous models by considering vegetation influences (Hashimoto et al. 2015), it used fixed parameters (e.g., basic root respiration rate and temperature sensitivity) without considering their potential variation under extreme climatic/environmental conditions. These structural simplifications and parameterization choices likely contribute to the observed discrepancies between model simulations and field measurements.

### Temporal variation of heterotrophic respiration and driving mechanisms

Our results showed a clear seasonal pattern in heterotrophic respiration with one peak in June or July among three sites. The seasonal amplitude of heterotrophic respiration was largest at EBF-N with a much higher level of SOC (30.9 g kg^−1^). Similarly, Zeeshan et al. (2022) reported a larger seasonality of heterotrophic respiration at the site with higher SOC level in subtropical forests of southeast China. For those low-SOC sites, our and other studies suggested that the weaker seasonality of soil temperature was largely responsible for a weaker seasonality of heterotrophic respiration.

We also found a considerable inter-annual variation in heterotrophic respiration at all three sites, which was caused by inter-annual variability of soil temperature particularly in winter and at the two northern sites with relative lower temperature (Table S5). Limited field studies (3–6 years) also reported considerable inter-annual variations of heterotrophic respiration in subtropical and temperate forests (Han et al. 2018; Teramoto et al. 2018; Wu et al. 2016), showing relatively higher rates of heterotrophic respiration in relatively warmer years (Huang et al. 2016; Teramoto et al. 2018; Yu et al. 2015). Modeling analysis demonstrated that there was a significant positive correlation between heterotrophic respiration and air temperature in the region of 20°–75° N over the past few decades, with a stronger correlation in higher latitudes (Nissan et al. 2023; Tang et al. 2020). All these analysis suggested that there might be inter-annual variation of heterotrophic respiration due to temperature change especially in low-temperature environment/regions. Apart from the effects of temperature, our and other studies showed that soil moisture also had considerable influence on the inter-annual variations of heterotrophic respiration in some regions with low rainfall and/or high evaporation (Lima et al. 2023; Zeeshan et al. 2022).

This study showed that winter rates of heterotrophic respiration were affected by ENSO events only at the southern site ENF-S, showing considerable response to both El Niño (7.9% increase) and La Niña events (8.0% decrease) (Table S7). There was also evidence for ENSO impacts on total soil respiration in tropical regions, showing higher rates during/following El Niño events (Cavaleri et al. 2017; Rubio and Detto 2017). An earlier study reported a decrease of total soil respiration but an increase of GPP during La Niña events in some parts of tropical and subtropical regions (Jones et al. 2001), indicating a potential reduction of heterotrophic respiration during La Niña. These analyses suggest that ENSO may play a role in regulating the inter-annual variability of soil heterotrophic respiration in some tropical and subtropical forests.

### Temporal variation of autotrophic respiration and driving mechanisms

This study showed strong seasonality of soil autotrophic respiration, with the highest rates in July at all three sites. The seasonal amplitude was greater at ENF-N than at EBF-N and ENF-S, which corresponded to the much larger seasonality of FRB at ENF-N (325 vs. 125–172 g C m^−2^). A previous study found that the Q_10_ value for autotrophic respiration was positively correlated with FRB over a seasonal cycle at temperate forest (Han and Jin 2018), also indicating the dominant role of FRB on the seasonality of autotrophic respiration.

Our results revealed considerable inter-annual variability in autotrophic respiration that showed no clear response to ENSO events across three selected sites. This finding aligns with satellite-based observations demonstrating weak correlations between ENSO and tree growth indicators (e.g., GPP, NDVI) in certain tropical/subtropical regions (Jones et al. 2001; Yan et al. 2021). The apparent resilience of autotrophic respiration to ENSO was further supported by global-scale modeling studies. For instance, the VEGAS model simulations (Qian et al. 2008) estimated that ENSO events drive a 25% variation in heterotrophic respiration but only a 7% change in autotrophic respiration, suggesting that plant respiration may be more buffered against climate anomalies than microbial decomposition processes. Synthesizing soil respiration observations across East Asia, Shen et al. (2024) found consistently weaker correlations for autotrophic respiration with ONI compared to heterotrophic respiration across tropical, subtropical, warm-temperate, and mid-temperate zones. This differential response between soil heterotrophic and autotrophic respiration implied that vegetation might possess greater adaptive capacity to climatic disturbances compared to soil microbial communities (Sun et al. 2019; Zheng et al. 2021). Limited field studies also revealed a considerable inter-annual variation in autotrophic respiration in forests (Han et al. 2018; Tang et al. 2021), which was related to changes in root biomass (in warm zone) and/or environmental conditions (in cold zone) (Kukumagi et al. 2017; Yu et al. 2015; Zhang et al. 2021). A 3-year field study showed higher rates of autotrophic respiration corresponding to higher temperature in summer in cold forests (Kukumagi et al. 2017). However, some field studies (2–5 years) in subtropical forestland indicated that inter-annual variation in autotrophic respiration was explained by FRB and solar radiation rather than soil temperature or moisture (Han et al. 2018; Yu et al. 2015; Zhang et al. 2021). Our analyses demonstrated that inter-annual variability of vegetation (e.g., FRB and GPP) made a much greater contribution (76–87%) to the change of autotrophic respiration than soil temperature (13–24%) in both broadleaf and needleleaf forests. Moreover, we found a larger contribution from GPP to the interannual variations of autotrophic respiration in broadleaf forests than in needle forests. This finding aligns with documented higher GPP in broadleaf forests (Cao et al. 2022; Yao et al. 2018) and reflects fundamental physiological differences: broadleaf species maintain larger photosynthetic surfaces and higher carboxylation rates (Hopkins et al. 2013), thereby requiring elevated ATP production through respiration to support enhanced metabolic activity. Concurrently, the greater carbon fixation in broadleaf forests provides amplified substrate supply for respiratory processes (Tang et al. 2005).

We found a significantly increasing trend of autotrophic respiration over 2002–2022 with a slope of 2.06–6.05 g C m^−2^ year^−1^, which was largely related to FRB and GPP rather than soil temperature (Fig. 10, Table S6). A limited number of studies reported a significantly similar increasing trend of autotrophic respiration in forests (Tang et al. 2021; Yan et al. 2022). Tang et al. (2021) demonstrated that an increasing trend of global autotrophic respiration was associated with the increase in temperature, precipitation, atmospheric CO_2_ concentration, and nitrogen deposition. Supporting our results, satellite LAI in China’s forestlands also exhibited a significant increasing trend (i.e., greening) over recent decades (Forzieri et al. 2017; Piao et al. 2015), which was attributed to various driving factors/mechanisms (Chen et al. 2019). Climate change (such as warming) was responsible for the increasing trend of LAI in high latitudes/altitudes, whereas CO_2_ fertilization and land use change were the dominant factors in the subtropical region (Zhu et al. 2016). Apparently, there might be considerable differences over space and time in terms of the driving mechanisms for the increasing trend (including inter-annual variation) of autotrophic respiration in forests. Our results demonstrate that the temporal variation of autotrophic respiration was largely regulated by tree growth rather than climate condition in low-latitude forests.

### Differences between heterotrophic respiration and autotrophic respiration

Our results revealed a considerable difference in seasonality between heterotrophic respiration and autotrophic respiration. Specifically, autotrophic respiration exhibited much stronger seasonality and a greater contribution to total soil respiration during summer. This pronounced summer autotrophic respiration peak reflected accelerated belowground carbon allocation to root growth and maintenance respiration during peak photosynthetic activity, consistent with known plant phenological strategies (Jiao and Wang 2019; McCloskey et al. 2021).

This study showed that both heterotrophic respiration and autotrophic respiration exhibited the strongest interannual variations in the winter season, coinciding with substantial interannual fluctuations in soil temperature (0–5 °C) and root biomass dynamics under cold conditions (Tables S9, 10). The enhanced winter sensitivity aligns with established physiological principles: both microbial decomposers and plants demonstrate heightened temperature responsiveness near their lower thermal tolerance limits (Hartley et al. 2007; Liu et al. 2016; Monson et al. 2006). There was a larger inter-annual variation in autotrophic respiration than in heterotrophic respiration, which was also found in some temperate forests (Han et al. 2016, 2018). Limited field studies demonstrated large differences in inter-annual variability between heterotrophic respiration and autotrophic respiration in subtropical forests. For example, early reports showed lowest heterotrophic respiration and highest autotrophic respiration in the same year with the lowest temperature (Huang et al. 2016; Liu et al. 2015), and lowest rates in the year with the lowest soil moisture for both heterotrophic respiration and autotrophic respiration were found by Yu et al. (2015). Both our finding and the analysis by Han et al. (2018) indicated that the weaker inter-annual variation of heterotrophic respiration was due to the relatively smaller variation in soil temperature (Fig. S6a). The stronger inter-annual variation in autotrophic respiration could be explained by a greater variation in vegetation growth (e.g., fine root biomass and NDVI) based on our (Fig. S6b) and Yan et al. (2022)’ study.

We found a significantly increasing trend in autotrophic respiration (thus its contribution to total soil respiration), but no clear trend in heterotrophic respiration over 2002–2022. The increasing contribution of autotrophic respiration to total soil respiration suggests the increasing net carbon sink in subtropical forests of China, consistent with findings from some satellite-driven studies (Noormets et al. 2021; Pan et al. 2024). A field study in temperate forestlands (Yan et al. 2022) reported similar autotrophic respiration trends (Yan et al. 2022). However, contrasting our findings, two modeling studies indicated an increasing trend for both heterotrophic and autotrophic respiration in temperate–subtropical regions post-1980s (Tang et al. 2019, 2020). The increasing trend of autotrophic respiration was due to forest greening (driven by CO_2_ fertilization, land use change, and nitrogen deposition) over the past few decades (Chen et al. 2019; Zhu et al. 2016). Conversely, the absence of an increasing heterotrophic respiration trend in our and Yan et al. (2022)’s study related to the non-significant trend in soil temperature in temperate–subtropical forests of China after 2000 (Fig. S6). This stagnant temperature trend was also noted in a satellite-based study over 2003–2017 (Zhao et al. 2020a). In addition, a modeling study reported a decreasing trend in ecosystem respiration but a weakly increasing trend in GPP after 1998, associated with a ‘warming hiatus’ (Ballantyne et al. 2017), suggesting a possible downward trend in heterotrophic respiration. Given the differences in the relative contributions of heterotrophic respiration and autotrophic respiration in forests over time and space, it is essential to employ advanced models with realistic parameterizations of both heterotrophic respiration and autotrophic respiration to improve the estimations of carbon sources/sinks and to better understand the carbon cycle in terrestrial ecosystem.

## Conclusion

Independent models were developed and validated for both soil heterotrophic respiration and autotrophic respiration in the subtropical forests of southeast China. Rates of heterotrophic respiration and autotrophic respiration were estimated over 2002–2022 at three representative sites, using validated independent models. Our results showed considerable differences between heterotrophic respiration and autotrophic respiration in terms of magnitude, seasonality, inter-annual variability and change trend. There were smaller seasonal and inter-annual variations in heterotrophic respiration than autotrophic respiration at all three sites, although the contribution of heterotrophic respiration was greater than that of autotrophic respiration. The temporal variation of heterotrophic respiration was mainly regulated by soil temperature, with soil moisture as the secondary influencing factor, whereas the driving factors for autotrophic respiration were vegetation growth-related variables, e.g., fine root biomass and GPP. There was a significant increasing trend in autotrophic respiration (but not in heterotrophic respiration), which was attributed to the significant increase in fine root biomass and GPP rather than soil temperature. ENSO events had no clear influence on inter-annual variation of autotrophic respiration at the three sites but some influences on heterotrophic respiration at the southern site. The significant differences between soil heterotrophic respiration and autotrophic respiration highlight the necessity and importance of separately modeling and estimating the two components of soil respiration and evaluating their responses to changing climate in forests. Our independent models provide valuable mechanistic insights for improving soil carbon modules in terrestrial carbon cycle models.

## Supplementary Information

Additional file 1.

## Data Availability

All model forcing data are publicly available at the links provided. Soil respiration data (model outputs) are available at https://doi.org/10.5281/zenodo.14177121.
