# Supplementary material for: Seasonal and interannual variations of soil heterotrophic respiration and autotrophic respiration in subtropical forests of southeast China: independent process-based models
Source: Geosci Lett. 2025 Jun 21;12(1):27. doi: 10.1186/s40562-025-00399-1 (PMC12182501; doi:10.1186/s40562-025-00399-1)
Supplement: Supplementary file 1 — Additional file 1. [file 40562_2025_399_MOESM1_ESM.docx]

**Seasonal and interannual variations of soil heterotrophic respiration and autotrophic** **respiration in subtropical forests of southeast China: independent process-based models**

Yibo Yan^a,b^, Xiujun Wang^a*^, Georg Wohlfahrt^b^, Ni Huang^c^

^a^ Faculty of Geographical Science, Beijing Normal University, Beijing, 100875, China

^b^ Universität Innsbruck, Institut für Ökologie, Innsbruck, 6020, Austria

^c^ State Key Laboratory of Remote Sensing Science, Aerospace Information Research Institute, Chinese Academy of Sciences, Beijing, 100094, China

^*^ Corresponding author: Xiujun Wang (xwang@bnu.edu.cn)


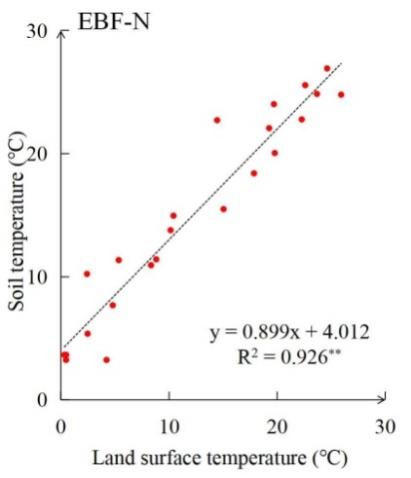

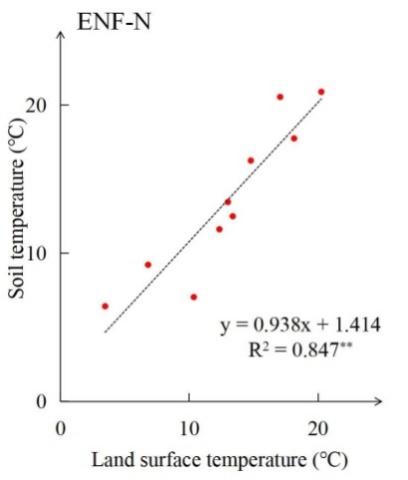

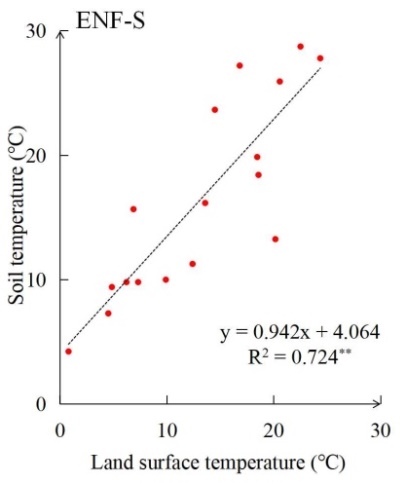


**Fig. S1.** Relationship between land surface temperature at night-time and soil temperature at three sites. Two asterisks indicate the significance at p < 0.01.


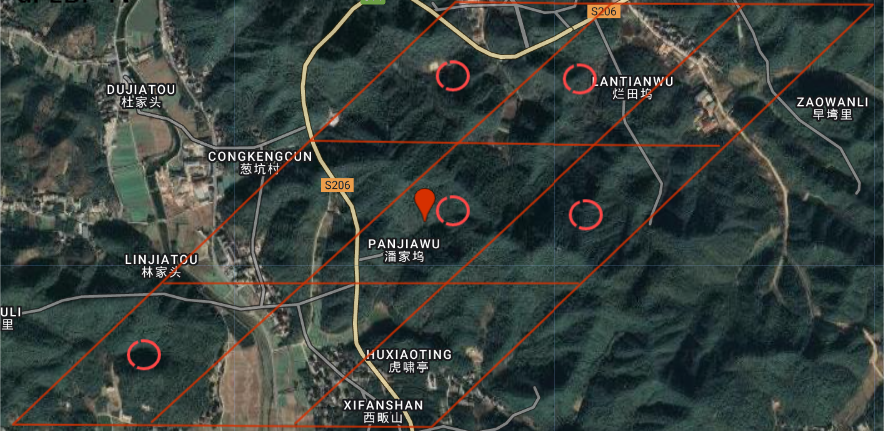


**Fig. S2**. Spatial distribution of forest cover and qualified pixels (marked by red circles) within the 3×3 pixel window at EBF-N site.


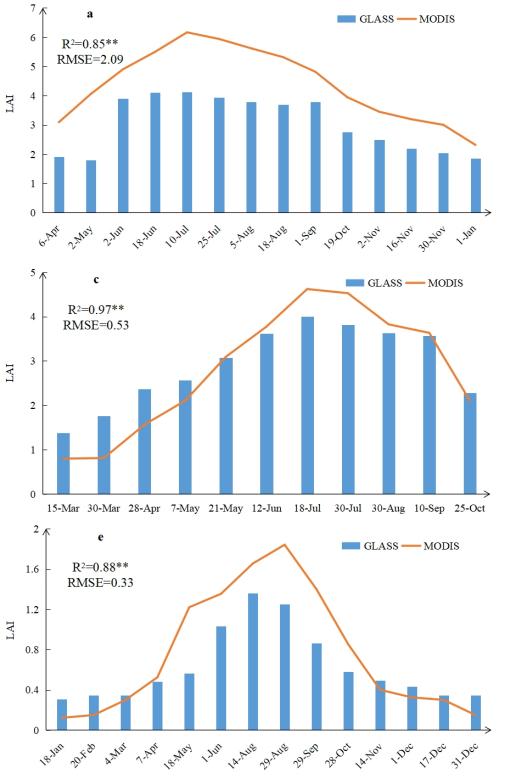

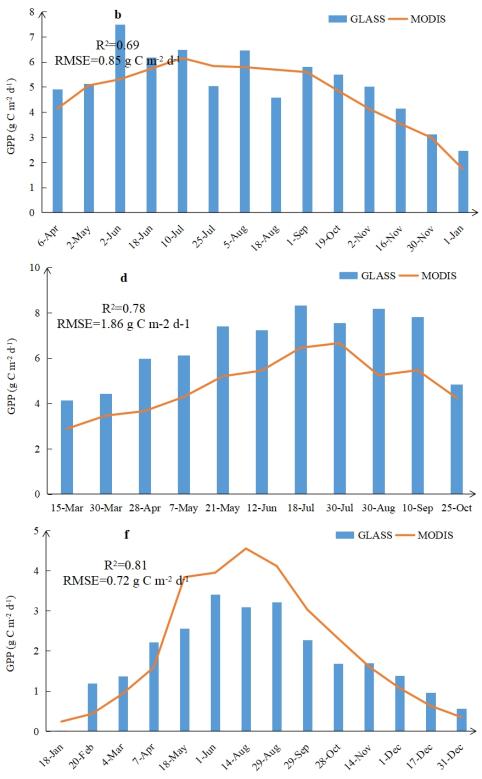


**Fig. S3.** The seasonal variation of GLASS LAI (bars) and MODIS LAI (line) at (a) EBF-N, (c) ENF-N and (e) ENF-S; The seasonal variation of GLASS GPP (bars) and MODIS GPP (line) at (b) EBF-N, (d) ENF-N and (f) ENF-S.


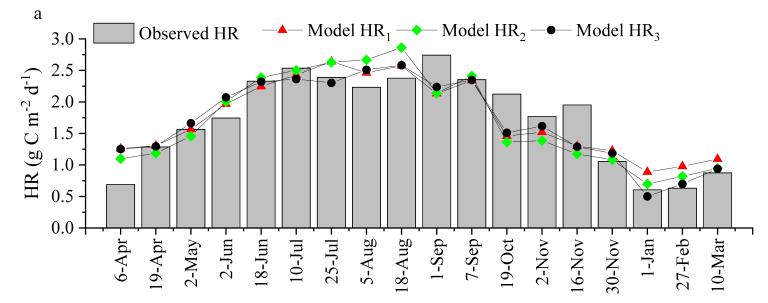

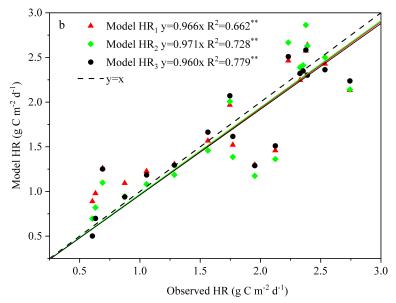

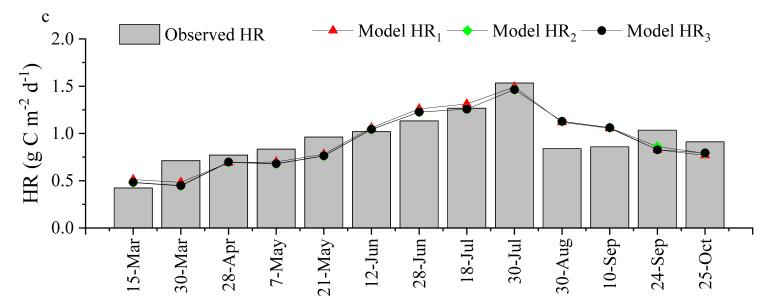

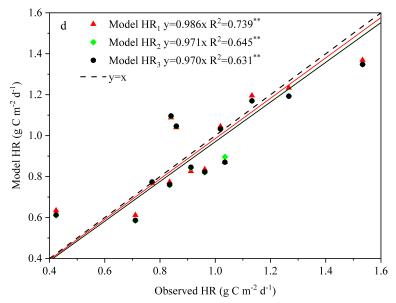

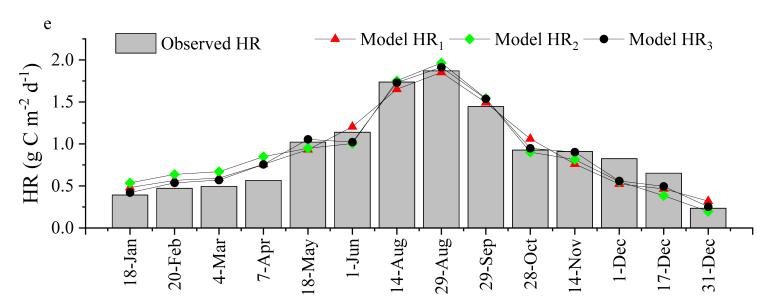

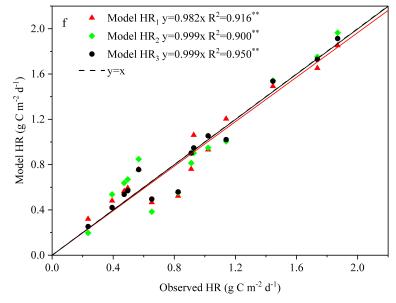


**Fig. S4.** Seasonal variation of observed (bars) and model (dots) heterotrophic respiration (HR) using three models at (a) EBF-N, (c) ENF-N and (e) ENF-S; Correlation between observed and model HR at (b) EBF-N, (d) ENF-N and (f) ENF-S. Two asterisks indicate the significance at p < 0.01.


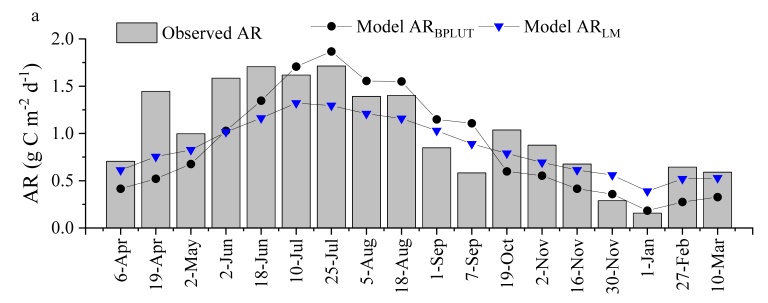

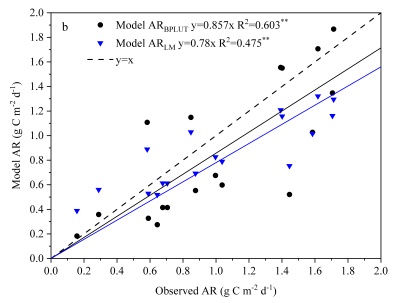

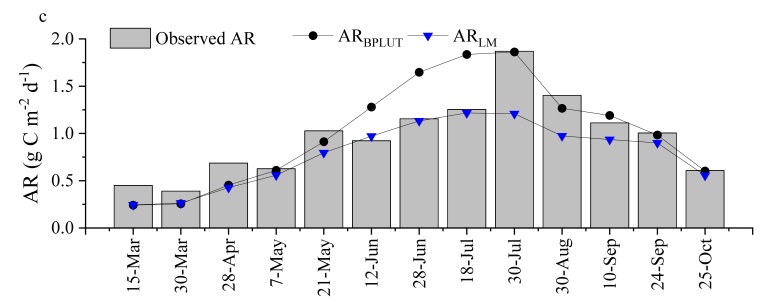

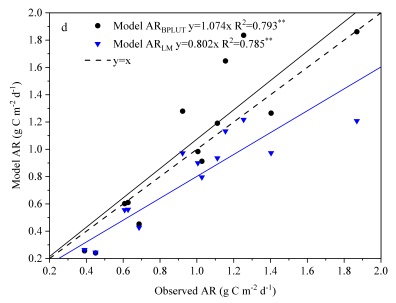

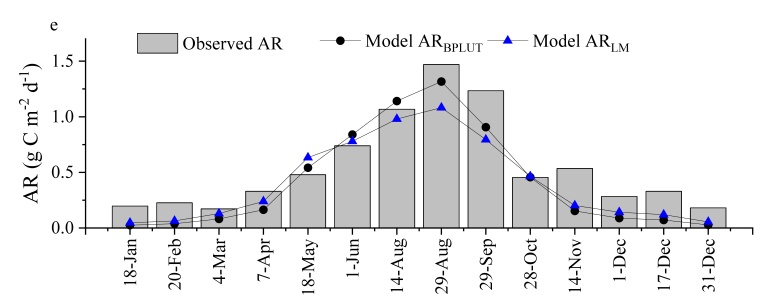

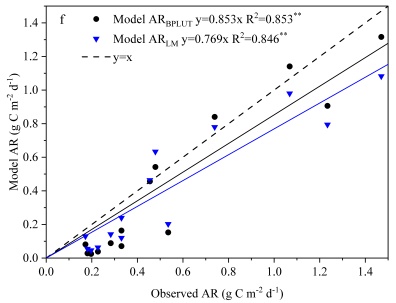


**Fig. S5.** Seasonal variation of observed (bars) and model (dots) autotrophic respiration (AR) using three models at (a) EBF-N, (c) ENF-N and (e) ENF-S; Correlation between observed and model AR at (b) EBF-N, (d) ENF-N and (f) ENF-S. Two asterisks indicate the significance at p < 0.01.


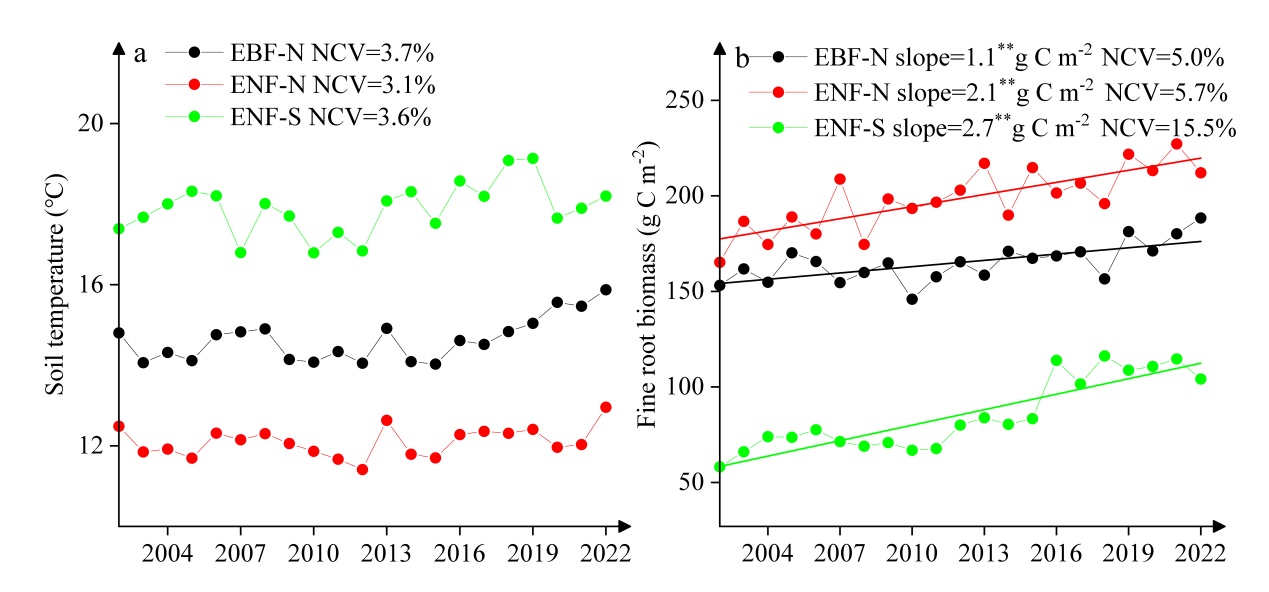


**Fig. S6.** The inter-annual variations of annual mean in (a) soil temperature and (b) fine root biomass over 2002-2022. Straight lines denote a significant increasing trend, and slopes annual change rates. Two asterisks indicate the significance at p < 0.01.

**Table S1**

Statics relationship between field soil temperature (T) and moisture (M) and satellite derived land surface temperature (LST) and soil moisture (SM).

| Sites | Temperature | Moisture |
| --- | --- | --- |
| EBF-N | $T=0.899\cdot LST+4.012$ | $M=0.96\cdot\mathrm{SM}$ |
| ENF-N | $T=0.938\cdot LST+1.414$ | $M=1.01\cdot\mathrm{SM}$ |
| ENF-S | $T=0.420\cdot LST+4.064$ | $M=0.45\cdot\mathrm{SM}$ |

**Table S2**

Evaluation of three HR models at three sites. Two asterisks indicate the significance at p < 0.01.

|  | EBF-N | | | ENF-N | | | ENF-S | | |
| --- | --- | --- | --- | --- | --- | --- | --- | --- | --- |
|  | HR_1_ | HR_2_ | HR_3_ | HR_1_ | HR_2_ | HR_3_ | HR_1_ | HR_2_ | HR_3_ |
| Slope | 0.966 | 0.971 | 0.960 | 0.986 | 0.971 | 0.970 | 0.983 | 0.999 | 0.999 |
| R^2^ | 0.66^**^ | 0.73^**^ | 0.78^**^ | 0.74^**^ | 0.64^**^ | 0.63^**^ | 0.92^**^ | 0.90^**^ | 0.95^**^ |
| RMSE | 0.344 | 0.372 | 0.309 | 0.135 | 0.136 | 0.139 | 0.136 | 0.160 | 0.110 |
| NSD | 0.836 | 1.016 | 0.918 | 0.866 | 0.855 | 0.854 | 0.967 | 1.029 | 1.018 |
| AIC | -32.4 | -31.6 | -38.3 | -46.1 | -47.8 | -47.4 | -49.9 | -47.3 | -57.9 |

**Table S3**

Evaluation of two parameterized methods for AR model at three sites. Two asterisks indicate the significance at p < 0.01.

|  | EBF-N | | ENF-N | | ENF-S | |
| --- | --- | --- | --- | --- | --- | --- |
|  | AR_BPLUT_ | AR_LM_ | AR_BPLUT_ | AR_LM_ | AR_BPLUT_ | AR_LM_ |
| Slope | 0.857 | 0.780 | 1.074 | 0.802 | 0.853 | 0.769 |
| R^2^ | 0.60^**^ | 0.48^**^ | 0.79^**^ | 0.78^**^ | 0.86^**^ | 0.85^**^ |
| RMSE | 0.373 | 0.322 | 0.258 | 0.256 | 0.194 | 0.212 |
| NSD | 1.112 | 0.593 | 1.352 | 0.818 | 1.079 | 0.883 |
| AIC | -31.51 | -36.82 | -31.25 | -31.4 | -41.97 | -39.50 |

**Table S4**

The selected model/parameterized methods at three sites.

| HR/AR | Sites | Model |
| --- | --- | --- |
| HR_3_ | EBF-N | $HR=2.16min(\frac{T}{T+13.5} , \frac{M}{M+17.5}){\cdot e}^{0.0236T}$ |
|  | ENF-N | $HR=0.68min(\frac{T}{T+10.4} , \frac{M}{M+16}){\cdot e}^{0.0594T}$ |
|  | ENF-S | $HR=0.925min(\frac{T}{T+14} , \frac{M}{M+13}){\cdot e}^{0.039T}$ |
| AR_BPLUT_ | EBF-N | $\mathrm{AR}=0.8\cdot\frac{LAI}{0.0259}\cdot0.00519\cdot2^{(T-20)/10}+0.03\cdot GPP$ |
|  | ENF-N | $AR=0.96\cdot\frac{LAI}{0.0141}\cdot0.00519\cdot2^{(T-20)/10}+0.03\cdot GPP$ |
|  | ENF-S |  |

**Table S5**

Statistics of heterotrophic and autotrophic respiration and their driving factors over seasonal cycle at three sites. Coefficient of variation (C.V.) was calculated by dividing the standard deviation (S.D.) by the annual mean.

|  |  | July | January | Annual  mean | Summer-winter | S.D. | C.V. (%) |
| --- | --- | --- | --- | --- | --- | --- | --- |
| Soil temperature  (°C) | EBF-N | 24.9 | 4.9 | 14.6 | 18.1 | 7.2 | 49 |
|  | ENF-N | 20.0 | 3.5 | 12.1 | 15.3 | 6.0 | 50 |
|  | ENF-S | 28.3 | 6.4 | 17.9 | 20.0 | 7.8 | 44 |
| Soil moisture  (%) | EBF-N | 33.5 | 30.1 | 31.1 | 3.4 | 3.1 | 10 |
|  | ENF-N | 29.9 | 22.1 | 27.8 | 5.7 | 2.9 | 11 |
|  | ENF-S | 16.4 | 14.0 | 15.2 | 2.2 | 1.3 | 8 |
| Precipitation  (mm month^-1^) | EBF-N | 185 | 90 | 143 | 132 | 62 | 67 |
|  | ENF-N | 221 | 28 | 110 | 161 | 66 | 60 |
|  | ENF-S | 146 | 65 | 122 | 93 | 58 | 48 |
| FRB  (g C m^-2^) | EBF-N | 259 | 76 | 165 | 172 | 70 | 43 |
|  | ENF-N | 401 | 53 | 200 | 325 | 136 | 68 |
|  | ENF-S | 158 | 19 | 86 | 125 | 51 | 60 |
| GPP  (g C m^-2^ d^-1^) | EBF-N | 6.3 | 1.7 | 4.8 | 4.3 | 1.9 | 38 |
|  | ENF-N | 5.8 | 0.9 | 3.8 | 4.5 | 1.8 | 47 |
|  | ENF-S | 4.9 | 0.6 | 3.0 | 4.0 | 1.6 | 53 |
| Heterotrophic respiration  (g C m^-2^ d^-1^) | EBF-N | 2.5 | 0.6 | 1.6 | 1.7 | 0.6 | 42 |
|  | ENF-N | 1.5 | 0.2 | 0.8 | 1.2 | 0.5 | 56 |
|  | ENF-S | 1.6 | 0.4 | 1.0 | 1.1 | 0.4 | 43 |
| Autotrophic respiration  (g C m^-2^ d^-1^) | EBF-N | 1.7 | 0.2 | 0.8 | 1.4 | 0.5 | 70 |
|  | ENF-N | 2.0 | 0.1 | 0.8 | 1.7 | 0.7 | 85 |
|  | ENF-S | 1.3 | 0.05 | 0.5 | 1.1 | 0.5 | 83 |

**Table S6**

Statistics of heterotrophic and autotrophic respiration in different seasons over 2002-2022. Detrended standard deviation (DSD) is standard deviation with a significant linear trend removed. Normalized C.V. (NCV) is a product of DSD divided by detrended-mean. Slope is the annual change rate with a significant linear trend. One asterisk and two asterisks indicate the significance at p < 0.05 and at p < 0.01, respectively.

| Statistics | Season/annual | Heterotrophic respiration | | | Autotrophic respiration | | |
| --- | --- | --- | --- | --- | --- | --- | --- |
|  |  | EBF-N | ENF-N | ENF-S | EBF-N | ENF-N | ENF-S |
| Mean  (g C m^-2^ d^-1^) | Spring | 1.54 | 0.80 | 1.04 | 0.68 | 0.61 | 0.46 |
|  | Summer | 2.35 | 1.39 | 1.51 | 1.54 | 1.81 | 1.18 |
|  | Autumn | 1.61 | 0.79 | 1.00 | 0.70 | 0.71 | 0.46 |
|  | Winter | 0.73 | 0.26 | 0.44 | 0.19 | 0.13 | 0.07 |
|  | Annual | 1.56 | 0.81 | 1.00 | 0.78 | 0.81 | 0.54 |
| DSD  (g C m^-2^ d^-1^) | Spring | 0.09 | 0.05 | 0.07 | 0.07 | 0.07 | 0.06 |
|  | Summer | 0.06 | 0.07 | 0.08 | 0.07 | 0.14 | 0.18 |
|  | Autumn | 0.07 | 0.05 | 0.05 | 0.07 | 0.10 | 0.07 |
|  | Winter | 0.08 | 0.03 | 0.06 | 0.03 | 0.01 | 0.01 |
|  | Annual | 0.05 | 0.03 | 0.04 | 0.04 | 0.05 | 0.07 |
| NCV  (%) | Spring | 5.74 | 6.44 | 6.73 | 11.24 | 13.89 | 19.49 |
|  | Summer | 2.71 | 5.06 | 5.26 | 4.78 | 8.23 | 20.88 |
|  | Autumn | 4.25 | 6.03 | 5.17 | 11.25 | 14.69 | 23.57 |
|  | Winter | 10.64 | 11.56 | 13.35 | 17.77 | 13.50 | 40.94 |
|  | Annual | 2.97 | 3.73 | 4.24 | 5.35 | 6.65 | 18.28 |
| Slope  (g C m^-2^ yr^-1^) | Spring | / | / | / | 0.54^*^ | 1.07^**^ | 1.26^**^ |
|  | Summer | / | / | / | / | 1.47^**^ | 2.96^**^ |
|  | Autumn | / | / | / | 0.73^**^ | / | 1.41^**^ |
|  | Winter | / | / | / | 0.37^**^ | 0.18^**^ | 0.34^**^ |
|  | Annual | 1.38^**^ | / | / | 2.05^**^ | 3.25^**^ | 6.05^**^ |

**Table S7**

Detrended-means of heterotrophic respiration and autotrophic respiration in winter season for El Niño (n=7) La Niña (n=9) years and their relative changes. Detrended-mean is the mean with a significant linear trend removed. Relative change is calculated as the relative increase/decrease compared with the detrended-mean of all years or neutral years. The figures in parentheses indicate the number of El Niño or La Niña events.

|  | El Niño/  La Niña | Heterotrophic respiration | | | Autotrophic respiration | | |
| --- | --- | --- | --- | --- | --- | --- | --- |
|  |  | EBF-N | ENF-N | ENF-S | EBF-N | ENF-N | ENF-S |
| Detrended-mean  (g C m^-2^ d^-1^) | El Niño (7) | 0.729 | 0.253 | 0.471 | 0.157 | 0.105 | 0.036 |
|  | La Niña (9) | 0.726 | 0.253 | 0.402 | 0.155 | 0.107 | 0.035 |
|  | Neutral years(5) | 0.726 | 0.266 | 0.451 | 0.180 | 0.105 | 0.033 |
|  | Mean of all years (21) | 0.727 | 0.256 | 0.437 | 0.162 | 0.106 | 0.035 |
| Relative change to mean of all years (%) | El Niño(7) | 0.30 | -1.03 | 7.86 | -2.64 | -0.74 | 2.02 |
|  | La Niña (9) | -0.15 | -1.26 | -7.95 | -4.12 | 0.91 | 0.88 |
| Relative change to mean of neutral years (%) | El Niño(7) | 0.45 | -4.58 | 4.41 | -12.37 | -0.15 | 6.72 |
|  | La Niña (9) | 0.00 | -4.80 | -10.89 | -13.70 | 1.51 | 5.53 |

**Table S8**

The contribution (%) of influence factors to the changes of annual heterotrophic respiration and autotrophic respiration at three sites.

| Soil respiration | Factors | EBF-N | | ENF-N | | ENF-S | |
| --- | --- | --- | --- | --- | --- | --- | --- |
|  |  | Range | Mean | Range | Mean | Range | Mean |
| Heterotrophic respiration | Soil temperature | 100-100 | 100±0 | 100-100 | 100±0 | 8-94 | 51±28 |
|  | Soil moisture | 0-0 | 0±0 | 0-0 | 0±0 | 0-92 | 49±28 |
| Autotrophic respiration | Soil temperature | 4-55 | 24±16 | 2-39 | 16±10 | 3-23 | 13±6 |
|  | Fine root biomass | 17-91 | 54±16 | 53-90 | 77±10 | 57-95 | 78±8 |
|  | GPP | 5-61 | 22±16 | 1-17 | 6±4 | 1-25 | 9±5 |

**Table S9**

Person correlation coefficient (R) between heterotrophic respiration/autotrophic respiration and driving factors based on season and annual means, and changes relative to 2002. One, two and three asterisks indicate significance at p < 0.05 and p < 0.01, respectively.

| Factors | Season/annual | Heterotrophic respiration | | | Autotrophic respiration | | |
| --- | --- | --- | --- | --- | --- | --- | --- |
|  |  | EBF-N | ENF-N | ENF-S | EBF-N | ENF-N | ENF-S |
| Soil temperature | Spring | 0.999^**^ | 0.993^**^ | 0.907^**^ | 0.756^**^ | 0.354 | 0.343 |
|  | Summer | 0.777^**^ | 0.989^**^ | 0.722^**^ | 0.767^**^ | 0.426 | 0.675^**^ |
|  | Autumn | 0.972^**^ | 0.969^**^ | 0.786^**^ | 0.785^**^ | 0.186 | 0.495^*^ |
|  | Winter | 0.999^**^ | 1.000^**^ | 1.000^**^ | 0.593^**^ | 0.571^**^ | 0.364 |
|  | Annual mean | 0.967^**^ | 0.981^**^ | 0.900^**^ | 0.697^**^ | 0.348 | 0.688^**^ |
|  | Change to 2002 | 0.967^**^ | 0.981^**^ | 0.906^**^ | 0.578^**^ | 0.273 | 0.617^**^ |
| Soil moisture | Spring | -0.026 | -0.474^*^ | 0.488^*^ |  |  |  |
|  | Summer | 0.108 | 0.201 | 0.749^**^ |  |  |  |
|  | Autumn | -0.042 | -0.066 | 0.143 |  |  |  |
|  | Winter | -0.271 | 0.113 | -0.083 |  |  |  |
|  | Annual | -0.455^*^ | -0.212 | 0.328 |  |  |  |
|  | Change to 2002 | -0.519^*^ | -0.375 | 0.363 |  |  |  |
| Fine root biomass | Spring |  |  |  | 0.900^**^ | 0.950^**^ | 0.959^**^ |
|  | Summer |  |  |  | 0.565^**^ | 0.847^**^ | 0.983^**^ |
|  | Autumn |  |  |  | 0.926^**^ | 0.943^**^ | 0.962^**^ |
|  | Winter |  |  |  | 0.943^**^ | 0.886^**^ | 0.926^**^ |
|  | Annual |  |  |  | 0.905^**^ | 0.943^**^ | 0.983^**^ |
|  | Change to 2002 |  |  |  | 0.978^**^ | 0.996^**^ | 0.996^**^ |
| GPP | Spring |  |  |  | 0.745^**^ | 0.748^**^ | 0.899^**^ |
|  | Summer |  |  |  | 0.181 | 0.535^*^ | 0.907^**^ |
|  | Autumn |  |  |  | 0.668^**^ | 0.591^**^ | 0.842^**^ |
|  | Winter |  |  |  | 0.937^**^ | 0.822^**^ | 0.770^**^ |
|  | Annual |  |  |  | 0.715^**^ | 0.668^**^ | 0.923^**^ |
|  | Change to 2002 |  |  |  | 0.789^**^ | 0.813^**^ | 0.951^**^ |

**Table S10**

The coefficient of variation (C.V.) of soil temperature and fine root biomass in four seasons at three sites over 2002-2022.

| Variable | Season | EBF-N | ENF-N | ENF-S |
| --- | --- | --- | --- | --- |
| Soil temperature  (%) | Spring | 6.93 | 5.94 | 5.45 |
|  | Summer | 2.87 | 3.63 | 3.25 |
|  | Autumn | 5.11 | 5.04 | 5.32 |
|  | Winter | 12.53 | 11.90 | 13.86 |
| Fine root biomass  (%) | Spring | 11.09 | 13.25 | 20.32 |
|  | Summer | 3.39 | 7.55 | 16.96 |
|  | Autumn | 8.43 | 14.71 | 25.88 |
|  | Winter | 17.51 | 11.33 | 54.90 |
